# Restoration of SIRT3 Expression in Aged Mice Alleviates UUO‐Induced Renal Fibrosis by Reducing GSK‐3β Hyperacetylation

**DOI:** 10.1002/advs.202417248

**Published:** 2025-07-23

**Authors:** Jing Wang, Xiang Ren, Huan Lu, Zihao Guo, Xing Li, Yiqun Tian, Yisheng Yin, Zhenliang Qin, Kun Yun, Minglong Wu, Gang Chen, Xiaoyong Zeng

**Affiliations:** ^1^ Institute of Organ Transplantation Tongji Hospital, Tongji Medical College Huazhong University of Science and Technology Key Laboratory of Organ Transplantation Ministry of Education NHC Key Laboratory of Organ Transplantation Key Laboratory of Organ Transplantation Chinese Academy of Medical Sciences Wuhan Hubei 430030 China; ^2^ Department of Urology Xijing Hospital Fourth Military Medical University Xi'an Shaanxi 710038 China; ^3^ Division of Radiology Mayo Clinic Rochester MN 55901 USA; ^4^ Department of Obstetrics and Gynecology Tongji Hospital Tongji Medical College Huazhong University of Science and Technology Wuhan Hubei 430030 China; ^5^ Department of Urology Tongji Hospital Tongji Medical College Huazhong University of Science and Technology No.1095 Jiefang Avenue Wuhan Hubei 430030 China; ^6^ Department of Surgery Tongji Hospital Tongji Medical College Huazhong University of Science and Technology No.1095 Jiefang Avenue Wuhan Hubei 430030 China

**Keywords:** aging, deacetylation, kidney fibrosis, micelles, obstructive nephropathy, SIRT3

## Abstract

Aging increases the vulnerability of kidneys to injury and impairs their regenerative capacity. SIRT3 expression declines with aging and is associated with multiple age‐related pathologies. The expression profile and functional role of SIRT3 in renal aging remain unclear. Here, SIRT3 expression in aging kidneys is assessed and analyzed for its promoter methylation patterns using methylation‐specific PCR (MSP). It is found that aging exacerbates UUO‐induced renal fibrosis, associated with downregulated SIRT3 expression. Mechanistically, age‐related SIRT3 downregulation is mediated by hypermethylation of its promoter region. SIRT3 knockout exacerbated renal fibrosis in young mice subjected to UUO, whereas SIRT3 overexpression attenuated fibrosis in aged UUO mice. Integration of RNA‐seq and immunoprecipitation‐mass spectrometry (IP‐MS) analyses revealed that SIRT3 deficiency leads to hyperacetylation of GSK3β at lysine 15 (K15). This K15 hyperacetylation inhibited GSK3β activity, consequently stabilizing its substrate β‐catenin. Furthermore, self‐assembled PEG‐PCL‐PEG micelles are designed and synthesized to encapsulate hydrophobic honokiol (HKL). These micelles significantly enhanced the aqueous solubility and oral bioavailability of free HKL, maintained stable blood concentrations, and ultimately improved its anti‐fibrotic efficacy. These findings propose novel therapeutic strategies for managing renal fibrosis in the aging population and provide a foundation for developing new drugs and combination therapies.

## Graphical abstract

SIRT3 expression is significantly downregulated in the aged kidney, rendering the kidney more vulnerable to fibrotic changes in obstructive nephropathy. By promoting GSK‐3β deacetylation, SIRT3 indirectly inhibits Wnt/β‐catenin signaling, thereby suppressing renal fibrosis.

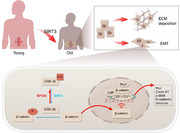

## 1 Introduction

The rapid global aging population has emerged as a prominent demographic trend, posing significant challenges to human health.^[^ ^1^ ^]^ Aging leads to diminished renal function and histological changes, making the kidneys more vulnerable to damage and impairing their recovery capacity. Specifically, kidney damage caused by ureteral obstruction in elderly patients is typically more severe and frequently fails to achieve functional recovery. This results in significant alterations in renal metabolism and glomerular filtration, as well as the progression of structural abnormalities within the kidney parenchyma, ultimately leading to the development of kidney fibrosis.^[^ ^2^, ^3^ ^]^ Despite significant progress in elucidating the mechanisms of renal fibrosis,^[^ ^4^ ^]^ there is still a translational gap between identifying potential therapeutic targets for age‐related renal fibrosis and translating this knowledge into clinical applications, such as anti‐aging strategies.

Sirtuin3 (SIRT3), an NAD^+^‐dependent protein deacetylase, is primarily localized within the mitochondria. Following the initial finding that Sirtuins (SIRTs) can enhance longevity of nematodes and flies, further research into the diverse functions of SIRT3 has deepened our comprehension of its roles in the mitochondrial metabolism, oxidative stress, autophagy, DNA repair, and inflammation.^[^ ^5^ ^]^ The expression of the SIRT3 has been observed to decrease with advancing age, and its deficiency has been explicitly associated with a variety of age‐related conditions, such as insulin resistance, cancer, and cardiovascular disorders.^[^ ^6^ ^]^ In the kidney, alterations in SIRT3 activity have been linked to various acute and chronic conditions, including acute kidney injury, diabetic nephropathy, and renal aging.^[^ ^7^, ^8^ ^]^ Several studies consistently reported a decline in the expression of SIRT3 during the aging process,^[^ ^9^, ^10^ ^]^ however, the molecular mechanisms responsible for the decline in SIRT3 expression are not yet fully elucidated. DNA methylation plays a crucial role in the progression of aging,^[^ ^11^ ^]^ as transcriptional suppression is commonly observed when CpG sites in gene promoters are excessively methylated, whereas demethylation facilitates gene transcription.^[^ ^12^ ^]^ However, to date, no direct studies have examined the methylation status of the SIRT3 gene in the context of renal aging.

Acetylation/deacetylation, a ubiquitous and crucial mechanisms of protein regulation, represent prominent post‐translational modifications (PTMs) with profound significance across various biological domains.^[^ ^13^ ^]^ Moreover, research into the renoprotective roles of SIRT3 in renal diseases has increasingly emphasized its deacetylase activity.^[^ ^14^ ^]^ According to a recent investigation, a mutation of the SIRT3 can provide a safeguard against acute kidney injury (AKI) by reducing the acetylation status of SOD2.^[^ ^15^ ^]^ Quan et al.^[^ ^16^ ^]^ discovered that SIRT3 regulates the NF‐κB/TGF‐β1 signaling pathway, and a subsequent study confirmed that SIRT3 promotes the deacetylation of β‐catenin at K49.^[^ ^17^ ^]^ However, the precise mechanisms by which SIRT3 alleviates renal fibrosis have not been fully elucidated. The Wnt/β‐catenin signaling pathway remains inactive in healthy adult kidneys, however, it becomes active again in different types of chronic kidney disease and plays a crucial part in facilitating the advancement of the illness.^[^ ^18^ ^]^ A recent investigation has indicated that inhibiting Wnt/β‐catenin/RAS signaling pathway can effectively prevent age‐related renal fibrosis.^[^ ^19^ ^]^ However, the precise target and regulatory mechanism by which SIRT3 modulates the Wnt signaling pathway remain to be fully elucidated. Glycogen synthase kinase‐3β (GSK3β) is a critical regulator within the Wnt/β‐catenin signaling pathway. As a central component, GSK3β phosphorylates β‐catenin, thereby targeting it for degradation.^[^ ^20^ ^]^ It has been documented that the activity and functionality of the GSK3β protein can be modulated through PTMs.^[^ ^21^ ^]^ So, in this study, we aimed to investigate the precise regulatory mechanism linking SIRT3 and the Wnt/β‐catenin pathway through the modulation of GSK3β.

Honokiol (HKL), a phenolic compound isolated from the traditional Chinese herb magnolia, exhibits multiple biological activities, including antitumor, antimicrobial, hepatoprotective, and cardioprotective effects.^[^ ^22^ ^]^ Notably, Vinodkumar et al.^[^ ^23^ ^]^ demonstrated that HKL upregulates SIRT3 expression. However, HKL's poor aqueous solubility (high hydrophobicity) has hindered its clinical translation. Previous studies have shown that the synthesized poly(ethylene glycol)‐poly(ε‐caprolactone)‐poly(ethylene glycol) (PEG‐PCL‐PEG) micelles are non‐toxic and can serve as a safe option for hydrophobic drug delivery systems.^[^ ^24^ ^]^ So, in this study, the HKL‐loaded PEG‐PCL‐PEG micelles were prepared and used to investigate its effect on renal fibrosis.

To summarize, in this study, we demonstrate that SIRT3 insufficiency is linked to more severe kidney fibrosis after ureteral obstruction in aging mice. SIRT3 deficiency promotes lysine acetylation of GSK3β, which in turn activates the Wnt/β‐catenin signaling pathway. In addition, the characteristics of HKL‐micelles were comprehensively evaluated as a promising delivery vehicle for HKL, demonstrating significant potential for clinical applications in the treatment of renal fibrosis. Our study provides further elucidation on the function of SIRT3 in inhibiting kidney fibrosis and uncover a novel mechanism dependent on acetylation that regulates GSK3β. These findings present novel therapeutic strategies for treating renal fibrosis in the aging population and establish a foundation for developing novel drugs and combination therapies.

## 2 Results

### 2.1 Aging Exacerbates Renal Injury and Fibrosis in UUO Mice

**Figure 1:**
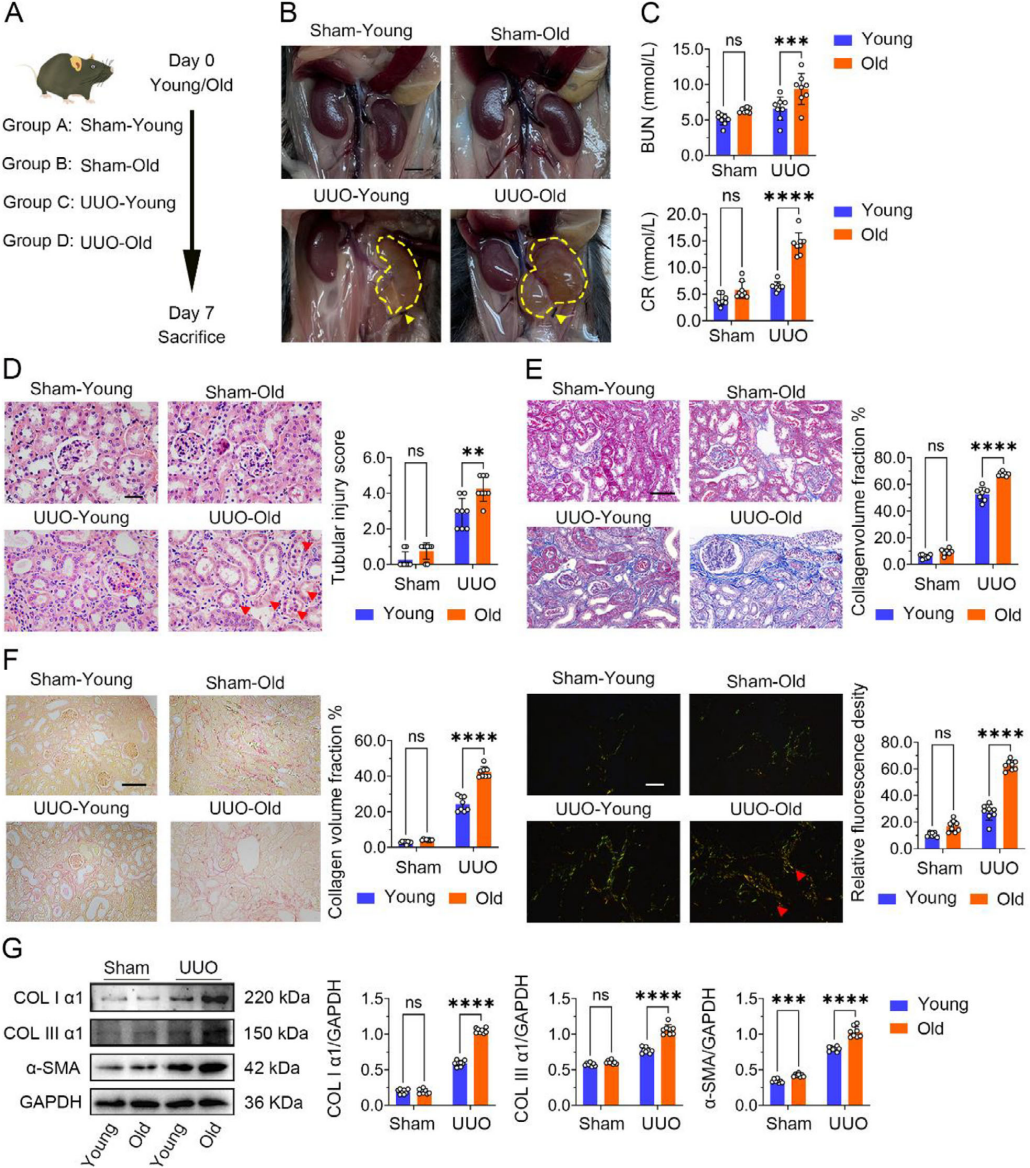
Aging aggravates renal fibrosis in UUO model mice. A) The schematic diagram illustrates the sequential steps of the animal procedure. Young, 6–8 weeks. Old, 22–24 months. UUO, unilateral ureteral obstruction. B) Macroscopic observation of the kidney. Scale bar, 10 mm. The yellow dashed line shows the outline of the kidney. C) Renal function tests. BUN, blood urea nitrogen. CR, creatinine. D) Representative kidney sections of mice stained with hematoxylin and eosin (H&E) with tubular injury score analysis. The red arrows indicate tubular atrophy, glomerulosclerosis, and tubular cell death. Scale bar = 50 µm. E) Masson's trichrome staining of kidney sections. Masson fibrotic area percentage presented and analysis. Scale bar = 50 µm. F) Sirius red staining of kidney sections were examined in light microscopy (left) or polarized light microscopy (right). Scale bar = 50 µm. The red arrow shows the I/III type collagen fibers. G) The western blot analyses showed the expression profiles of fibrotic markers (Collagen I α1 (COL1 α1), Collagen III α1 (COL3 α1), and α‐SMA) in renal tissues. The RM two‐way ANOVA (matched values are spread across a row) was conducted to compare the differences between groups. The RM two‐way ANOVA (matched values are spread across a row) was conducted to compare the differences between groups. 0.1234 (NS, no significance), 0.0332 (*), 0.0021 (**), 0.0002 (***), <0.0001 (****). 0.1234 (NS, no significance), 0.0332 (*), 0.0021 (**), 0.0002 (***), <0.0001 (****).

To investigate the impact of aging on the development of renal injury and fibrosis, we utilized mice of different age groups (young: 6–8 weeks; aged: 22–24 months) and subjected them to unilateral ureteral obstruction (UUO) or sham surgery. As shown in **Figure** **1**B, aged UUO mice exhibited more severe hydronephrosis and signs of ischemia compared to young UUO mice. Moreover, assessment of kidney function revealed significantly elevated levels of blood urea nitrogen (BUN) and serum creatinine (CR) in aged UUO mice compared to young UUO mice (Figure 1C). H&E staining revealed that kidneys from young sham‐operated mice exhibited normal tissue architecture, size, and glomerular morphology, with no signs of inflammatory cell infiltration (Figure 1D). Compared to sham‐operated controls, UUO induced significant glomerular sclerosis, tubular dilation, and a reduction in the number of normal glomeruli. Furthermore, comparing the two UUO groups, tubular sclerosis, atrophy, and edema were significantly more severe in aged UUO mice than in young UUO mice.

To clarify the degree of kidney fibrosis, renal sections were assessed with Masson's trichrome staining (Figure 1E). Masson's trichrome staining revealed significantly increased collagen deposition in the renal stroma and perivascular areas of UUO mice compared to sham‐operated mice (Figure 1E). Notably, this fibrotic deposition was markedly more pronounced in aged UUO mice than in young UUO mice. Sirius red staining further confirmed a significantly greater extent of renal interstitial fibrosis in aged UUO mice relative to young UUO mice (Figure 1F). Furthermore, Western blot analysis demonstrated significantly elevated protein expression of α‐smooth muscle actin (α‐SMA), collagen I, and collagen III in kidneys from aged UUO mice compared to young UUO mice (Figure 1G). Collectively, these results demonstrate that UUO induces renal dysfunction and fibrosis in mice, and this pathological response is significantly exacerbated by aging.

### 2.2 SIRT3 Exhibits Downregulation and Hypermethylation of Its Promoter in the Aging Kidney and Senescent HK‐2 Cells

**Figure 2:**
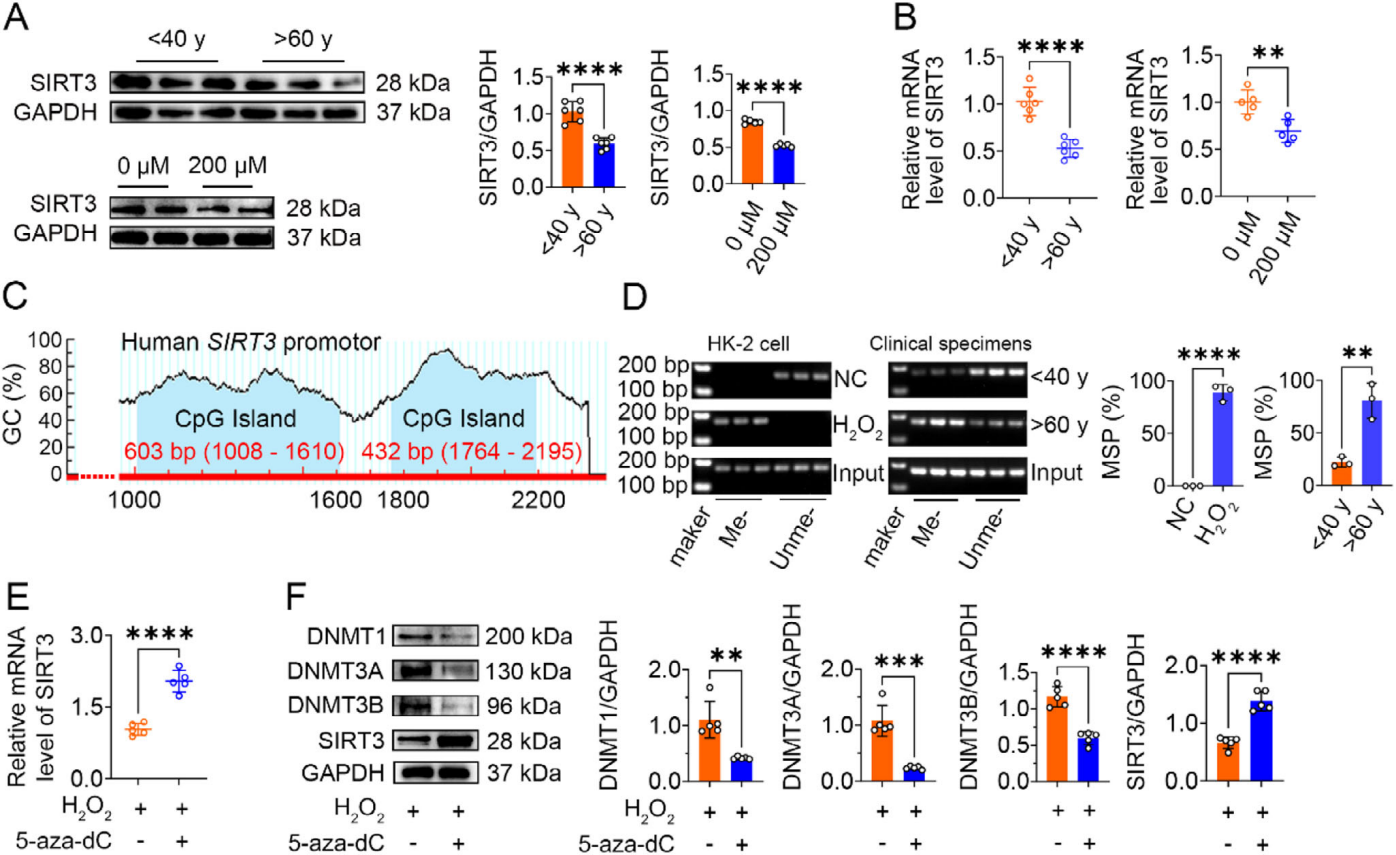
The decreased expression of SIRT3 during aging is attributed to DNA methylation modification. A) Western blots showing SIRT3 protein expression in clinical specimens (*n* = 6) or HK‐2 cells treated with H_2_O_2_. B) qRT‐PCR was showing SIRT3 mRNA expression level in clinical specimens or HK‐2 cells treated with H_2_O_2_. C) Schematic representations of human SIRT3 promoter regions are depicted, highlighting the presence of CpG islands (indicated in blue). D) Representative analysis of agarose gel for methylated (Me‐), unmethylated (Unme‐), and input PCR products. The quantitative analysis was presented accordingly, with normalization applied to the input PCR products. E) qRT‐PCR showed SIRT3 mRNA expression in senescent HK‐2 cells. F) Western blots showing SIRT3, DNMT1, DNMT3A, and DNMT3B protein expression in senescent HK‐2 cells. *p < 0.05, **p < 0.01, ****p < 0.0001. ns, no significance.

To investigate the role of SIRT3 in vivo and in vitro, we initially examined the levels of SIRT3 expression in kidney tissues of mice at different ages. qRT‐PCR analysis revealed a significant reduction in SIRT3 mRNA expression in the kidneys of aged mice compared to young mice (Figure S3A, Supporting Information). Subsequently, Western blot analysis and immunohistochemical staining confirmed significantly lower SIRT3 protein expression in the kidneys of aged mice compared to young mice (Figure S3B,C, Supporting Information). SIRT3 expression was also significantly downregulated in HK‐2 cells following treatment with H₂O₂ to induce senescence (**Figure** **2**A,B). Furthermore, consistent with the findings in murine and cellular models, SIRT3 expression was also significantly decreased in renal samples obtained from older human subjects compared to younger individuals (Figure 2A,B). These findings align with our observations from animal experiments, suggesting that the downregulation of SIRT3 potentially contributes significantly to the development of UUO‐induced kidney fibrosis.

We next investigated the underlying mechanism responsible for the age‐related downregulation of SIRT3 expression. MethPrimer (www.urogene.org/methprimer) analysis identified characteristic CpG islands within the SIRT3 promoter region, located approximately at positions 1008–1610 and 1764–2195 (relative to the transcription start site) (Figure 2C). MSP analysis demonstrated a significant increase in the methylation levels of the SIRT3 promoter in renal tissues from aged individuals compared to younger individuals, and in senescent HK‐2 cells compared to control cells (Figure 2D). Moreover, treatment with the DNA methyltransferase inhibitor 5‐aza‐2′‐deoxycytidine (5aza) reversed the downregulation of SIRT3 expression in senescent HK‐2 cells (Figure 2E,F). Therefore, our results preliminarily confirm that the age‐related decline in SIRT3 expression is likely attributable to hypermethylation of its promoter region, resulting in transcriptional repression.

### 2.3 SIRT3 Knockout Exacerbated Renal Fibrosis in Young UUO Mice, Whereas SIRT3 Overexpression Ameliorated UUO‐Induced Renal Fibrosis in Aged Mice

**Figure 3:**
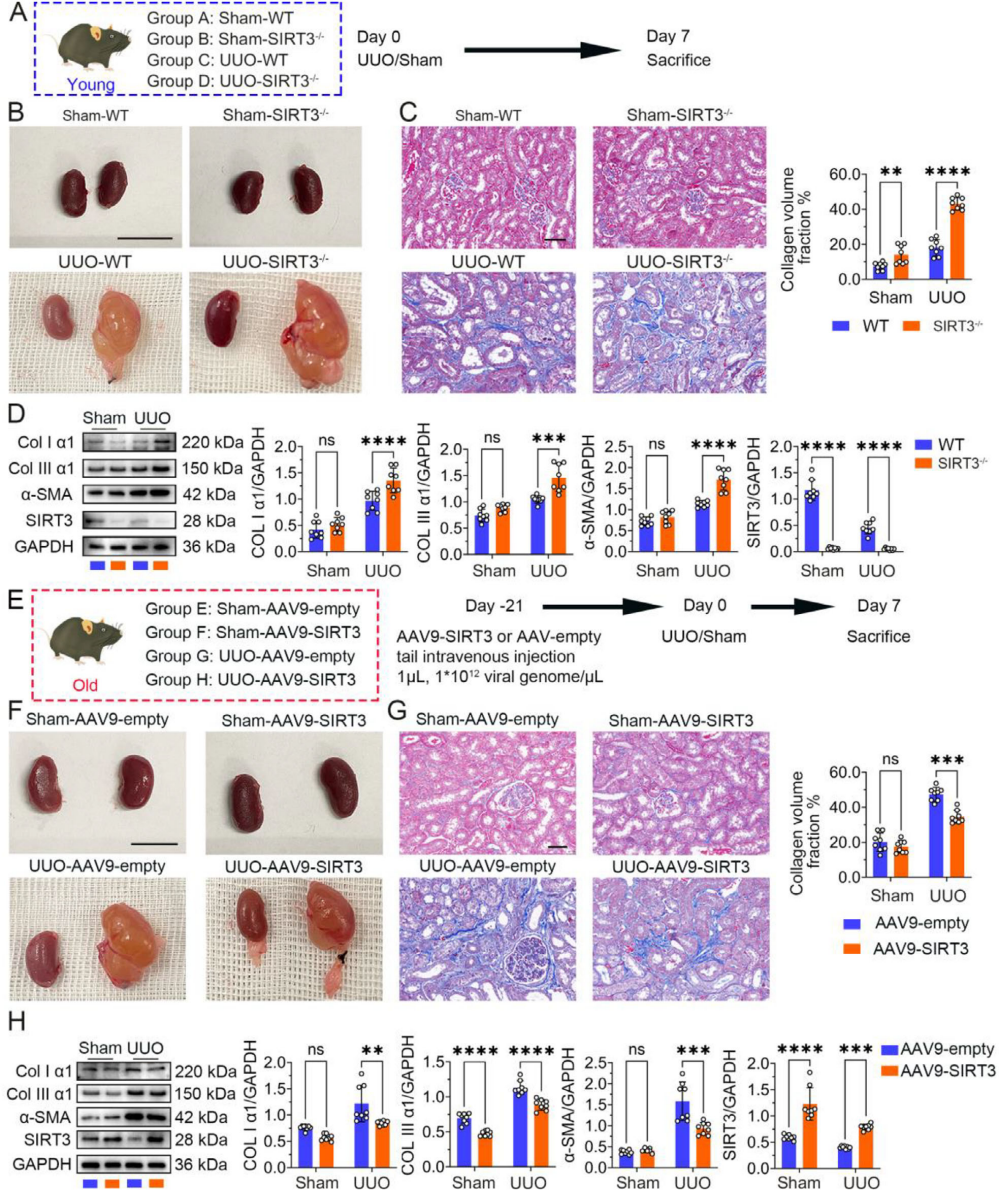
Intervention of SIRT3 expression can affect UUO‐induced renal fibrosis in mice of varying ages. A) The schematic diagram illustrates the sequential steps of the animal procedure. Young, 6–8 weeks. UUO, unilateral ureteral obstruction. B) Macroscopic observation of kidney tissues. Scale bar, 10 mm. C) Masson's trichrome staining of kidney sections. Scale bar = 50 µm. D) Western blots showing Collagen I α1, Collagen III α1, and α‐SMA protein expression. E) The schematic diagram illustrates the sequential steps of the animal procedure. Old, 22–24 months. UUO, unilateral ureteral obstruction. F) Macroscopic observation of kidney tissues. Scale bar, 10 mm. G) Masson's trichrome staining of kidney sections. Scale bar = 50 µm. H) Western blots showing Collagen I α1, Collagen III α1, α‐SMA, and SIRT3 protein expression. The RM two‐way ANOVA (matched values are spread across a row) was conducted to compare the differences between groups. 0.1234 (NS, no significance), 0.0332 (*), 0.0021 (**), 0.0002 (***), <0.0001 (****).

Our findings indicate that SIRT3 expression declines with aging (Sections 2.1 and 2.2), and this downregulation is associated with exacerbated renal fibrosis in UUO mice (Section 2.1). Consequently, we hypothesized that the age‐related reduction in SIRT3 expression is a key contributor to the promotion of UUO‐induced renal fibrosis. To directly validate the role of SIRT3 in renal fibrosis, we generated a UUO model using young mice with conditional knockout of SIRT3 specifically in renal tubular epithelial cells (Figure S1, Supporting Information). Compared to the WT UUO group, the kidneys of mice in the SIRT3^‐/‐^ UUO group exhibited more pronounced hydronephrosis (**Figure** **3**B). Subsequent histopathological and fibrotic assessments were performed. As shown in Figure 3C, kidneys from SIRT3^‐/‐^ UUO mice, which displayed severe hydronephrosis, showed increased extracellular matrix (ECM) deposition compared to WT UUO controls. WB analysis further confirmed a significant increase in the protein levels of key renal fibrosis markers in the SIRT3^‐/‐^ UUO group compared to the WT UUO group (Figure 3D).

To investigate the potential protective effect of SIRT3 overexpression on UUO‐induced renal fibrosis in aged mice, we transduced the kidneys of aged mice with recombinant adeno‐associated virus serotype 9 carrying SIRT3 (AAV9‐SIRT3) (Figure S4, Supporting Information). As depicted in Figure 3F, the UUO groups displayed noticeable enlargement and swelling of the kidneys, and a reduction in renal cortex thickness and significant dilation of the renal pelvis and calyces was observed upon incision along the kidney's coronal plane. There was a substantial increase in collagen fibrils of the kidney for the UUO group (Figure 3G). Following these pathological changes, a notable rise in protein levels of α‐SMA, Collagen I α1, and Collagen III α1 was observed within the UUO groups. Moreover, when SIRT3 was overexpressed in aged UUO mice, there was a significant decrease in fibrosis‐related markers that were upregulated by UUO (Figure 3H). These findings provide further evidence that increasing SIRT3 expression alleviates kidney injury and attenuates excessive extracellular matrix accumulation in aged UUO mice.

### 2.4 SIRT3 Inhibits EMT by Negatively Regulating the Wnt/β‐Catenin Signaling Pathway

To investigate the mechanism linking SIRT3 overexpression to protection against UUO‐induced renal fibrosis, as well as the underlying mechanisms, we transfected senescent HK‐2 cells with a pCDHMV‐SIRT3‐EF1‐copGFP‐T2A‐Puro plasmid (p‐SIRT3) to overexpress SIRT3. The transfection efficiency and SIRT3 overexpression were confirmed by qRT‐PCR and Western blot analysis, respectively (Figure S5, Supporting Information). As shown in Figure S5C (Supporting Information), treatment with transforming growth factor‐β (TGF‐β) induced characteristic morphological changes associated with EMT in senescent HK‐2 cells. In contrast, senescent HK‐2 cells overexpressing SIRT3 maintained a morphology similar to untreated control cells following TGF‐β stimulation. Notably, SIRT3 overexpression substantially downregulated the expression of the mesenchymal markers N‐cadherin and vimentin in TGF‐β‐treated senescent HK‐2 cells (Figure S5D,E, Supporting Information). These findings collectively demonstrate that SIRT3 overexpression effectively suppresses the TGF‐β‐induced EMT phenotype in senescent HK‐2 cells.

**Figure 4:**
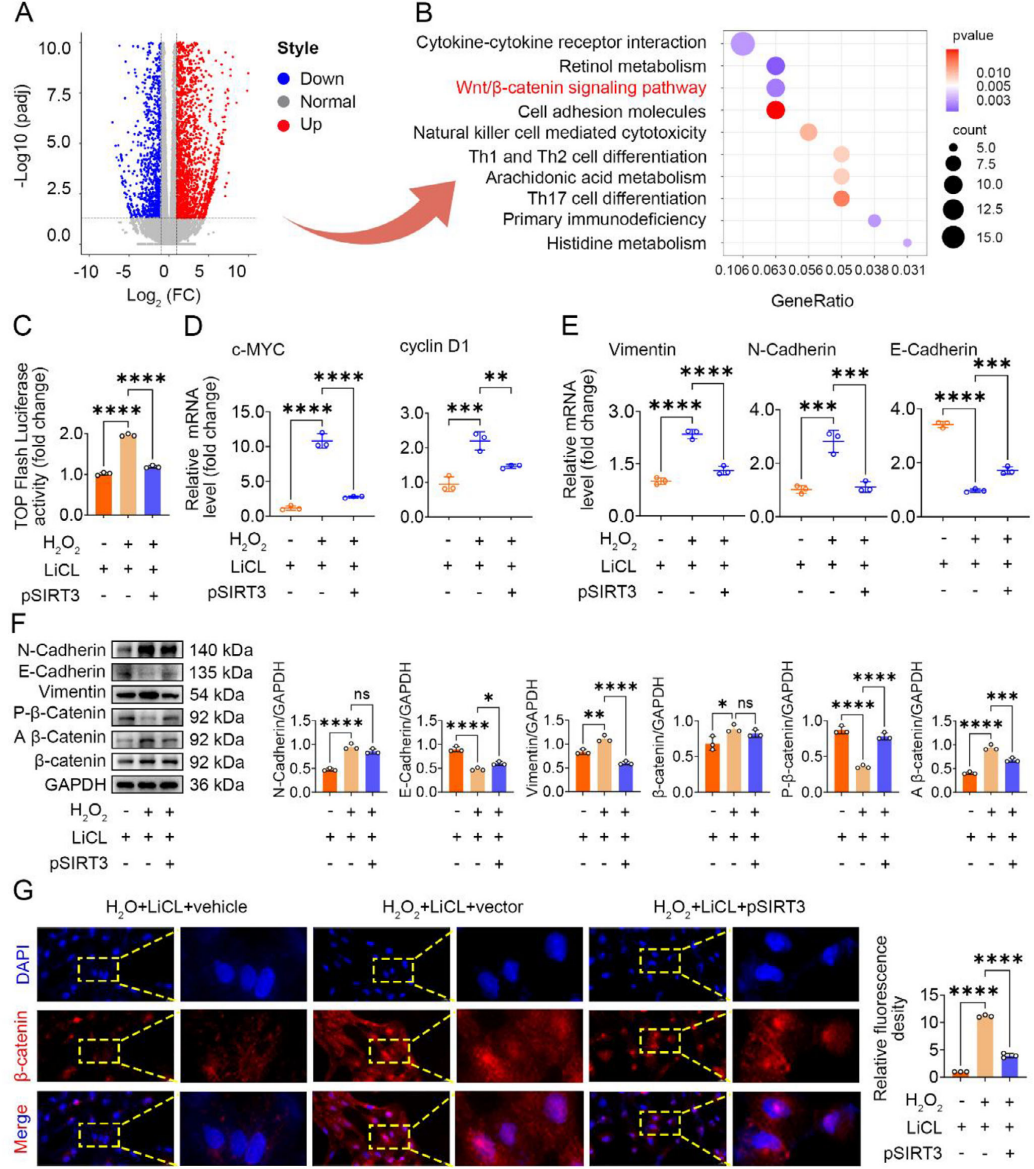
SIRT3 suppresses EMT through its negative modulation of the Wnt/β‐catenin signaling pathway. A) Volcano plot shows differentially expressed genes. UUO_Young vs UUO_Old. B) Conducting KEGG pathway enrichment analysis on the genes that exhibit differential expression. The top ten enriched pathways were shown. C) Top/Flash reporter activity assay. D) qRT‐PCR showed cMYC and cyclin D1. E) qRT‐PCR showing N‐cadherin, E‐Cadherin, and Vimentin mRNA expression. F) Western blots showing N‐cadherin, E‐Cadherin, Vimentin, β‐Catenin, Non‐phospho (Active) β‐Catenin‐S33/S37/T41, and Phospho‐β‐Catenin‐S552 protein expression. G) Immunofluorescence staining showing β‐Catenin protein expression. 500× magnification, 1000× magnification (enlarged). *p < 0.05, **p < 0.01, ***p < 0.001, ****p < 0.0001, ns, no significance.

To elucidate signaling pathways potentially associated with SIRT3 in the context of age‐exacerbated fibrosis, we performed RNA sequencing (RNA‐seq) on kidney tissues. RNA‐seq analysis revealed 3094 significantly upregulated and 2184 significantly downregulated genes in kidney tissues from aged UUO mice (Old_UUO) compared to young UUO mice (Young_UUO) (**Figure** **4**A,B). Kyoto Encyclopedia of Genes and Genomes (KEGG) pathway enrichment analyses of the differentially expressed genes suggested that the Wnt/β‐catenin signaling pathway was aberrantly activated in aged UUO mice compared to young UUO mice. Differential gene expression profiles between Sham_Young vs UUO_Young and Sham_Old vs UUO_Old groups are presented in Figure S6 (Supporting Information).

To validate the involvement of Wnt/β‐catenin signaling, we treated senescent HK‐2 cells with lithium chloride (LiCl), a known activator of Wnt signaling, as a positive control. LiCl treatment significantly increased the protein levels of active (non‐phosphorylated) β‐catenin (Figure 4F,G) and upregulated the mRNA expression of the Wnt target genes c‐MYC and cyclin D1 (Figure 4D). In contrast, overexpression of SIRT3 in LiCl‐treated senescent HK‐2 cells increased the levels of phosphorylated β‐catenin (targeted for degradation) (Figure 4F) and significantly reduced the mRNA expression of c‐MYC and cyclin D1 (Figure 4D). Furthermore, SIRT3 overexpression reduced TCF/LEF transcriptional reporter activity (Figure 4C) and inhibited LiCl‐induced nuclear translocation of β‐catenin (Figure 4G). Additionally, SIRT3 overexpression significantly suppressed the LiCl‐induced upregulation of vimentin and N‐cadherin and restored E‐cadherin expression in senescent HK‐2 cells (Figure 4E,F). These findings confirm that SIRT3 has an inhibitory effect on EMT by negatively regulating the Wnt/β‐catenin signaling pathway.

### 2.5 SIRT3 Mediates GSK‐3β Lysine Residue 15 Deacetylation to Promote β‐Catenin Phosphorylation

**Figure 5:**
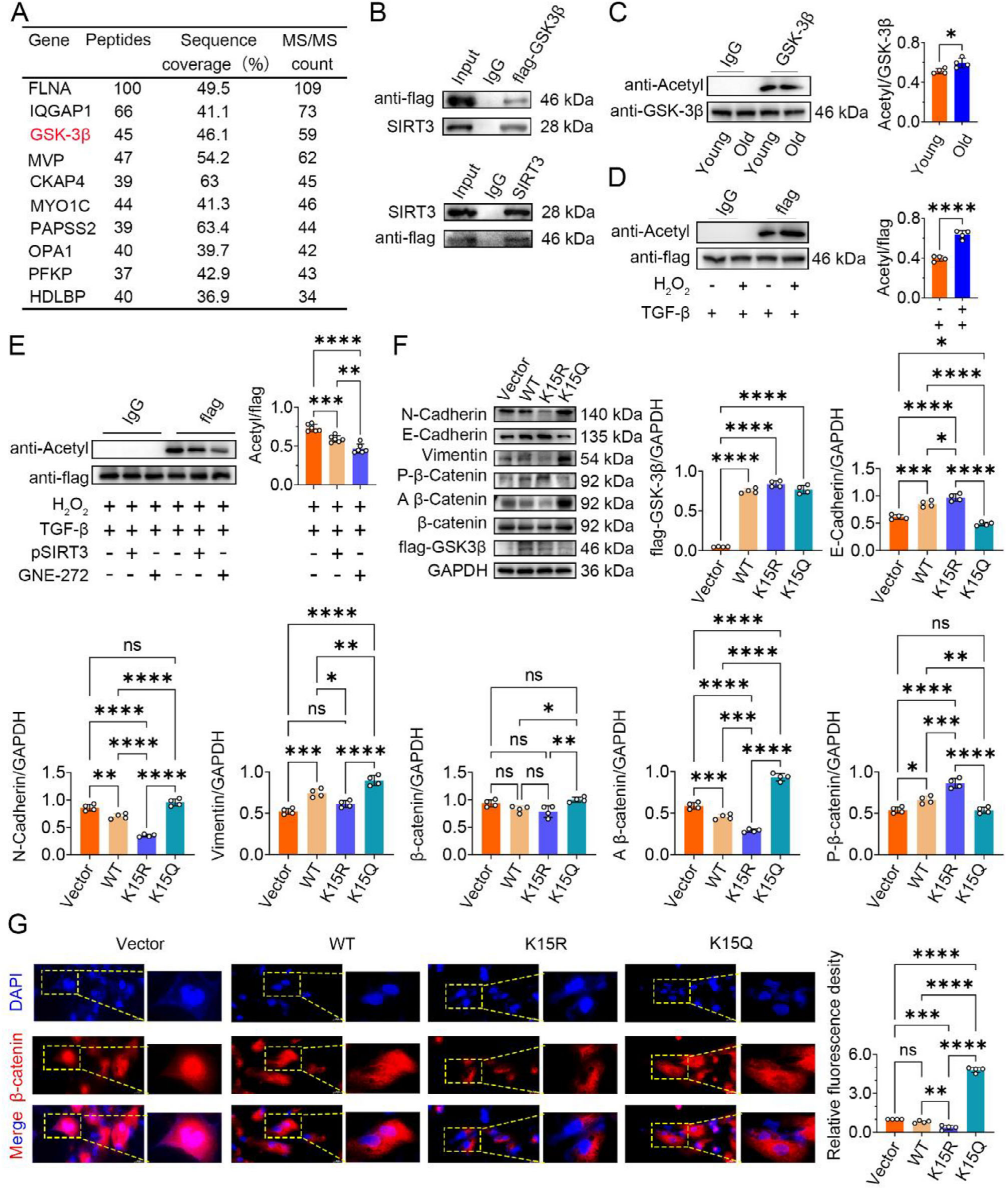
SIRT3 mediates GSK‐3β lysine 15 deacetylation to promote β‐catenin phosphorylation. A) IP‐MS identifies the proteins that interact with SIRT3. The top 10 proteins were shown. B) Interactions between SIRT3 and GSK3β were detected by Co‐IP assays. C,D) Western blots showing GSK3β protein acetylation level from kidney tissue and senescent HK‐2 cells, respectively. Flag‐GSK3β served as the standard. E) Western blots showing GSK3β protein acetylation level from senescent HK‐2 cells. Flag‐GSK3β served as the standard. F) Western blots showing flag‐GSK3β, β‐Catenin, non‐phospho (Active) β‐Catenin‐S33/S37/T41, Phospho‐β‐Catenin‐S552, N‐cadherin, E‐Cadherin, and Vimentin protein expression. G) Immunofluorescence staining showing β‐Catenin protein expression. 500× magnification, 1000× magnification (enlarged). *p < 0.05, **p < 0.01, ***p < 0.001, ****p < 0.0001, ns, no significance.

As SIRT3 is a NAD+‐dependent protein deacetylase, we investigated whether its protective effects against renal fibrosis are mediated through its deacetylase activity. Compared to young UUO mice, total lysine acetylation levels were significantly elevated in kidney tissues from aged UUO mice (Figure S7A, Supporting Information). Importantly, the acetylation level of GSK‐3β protein was also significantly higher in aged UUO mice than in young UUO mice (**Figure** **5**C). To identify potential SIRT3‐interacting proteins involved in Wnt signaling, we performed immunoprecipitation of SIRT3 followed by mass spectrometry (IP‐MS) analysis. The top 10 proteins included FLNA, IQGAP1, GSK‐3β, MVP, CKAP4, MYO1C, PAPSS2, OPA1, PFKP and HDLBP (Figure 5A). The interaction between SIRT3 and GSK‐3β in senescent HK‐2 cells was also confirmed by endogenous CoIP assay (Figure 5B). Moreover, total lysine acetylation levels (Figure S7B, Supporting Information) and the specific acetylation level of GSK‐3β (Figure 5D) were significantly elevated in H₂O₂‐treated senescent HK‐2 cells compared to untreated control cells. In TGF‐β and H₂O₂ co‐treated senescent HK‐2 cells, treatment with GNE‐272, an inhibitor of the acetyltransferase EP300, significantly decreased GSK‐3β acetylation levels, serving as a positive control (Figure 5E). Similar to the effect of GNE‐272, overexpression of SIRT3 also significantly reduced the acetylation level of GSK‐3β protein in TGF‐β and H₂O₂ co‐treated cells (Figure 5E).

Next, the amino acid sequence of GSK3β was retrieved from the NCBI database, and online prediction tools were utilized to identify potential acetylation sites. The results indicate that the 15th position lysine (K) of GSK‐3β is a site for acetylation modification, specifically targeted by the acetyltransferase EP300. (Figure S7C, Supporting Information). The COBALT tool analysis reveals that the 15th amino acid residue is conserved as lysine across multiple species, including humans, rhesus macaques, pigs, sheep, rabbits, mice, tropical clawed frogs, and zebrafish (Figure S7D, Supporting Information). This suggests a significant biological importance associated with potential mutations at this specific site during the evolutionary process.

To modulate the acetylation status of GSK3β at K15, we transfected senescent HK‐2 cells with mutant plasmids^[^ ^25^, ^26^ ^]^: GSK3β‐K15R (lysine to arginine mutation, mimicking a deacetylated state) and GSK3β‐K15Q (lysine to glutamine mutation, mimicking a constitutively acetylated state). Western blot analysis confirmed successful overexpression of wild‐type GSK3β (GSK3β‐WT) and the K15R and K15Q mutants in senescent HK‐2 cells (Figure 5F). Compared to GSK3β‐WT transfection, the GSK3β‐K15R mutant caused a more pronounced downregulation of N‐cadherin and vimentin and a stronger upregulation of E‐cadherin (Figure 5F). Conversely, the GSK3β‐K15Q mutant increased N‐cadherin and vimentin expression and decreased E‐cadherin expression relative to GSK3β‐WT transfection (Figure 5F). These findings indicate that GSK3β‐WT plasmid transfection can effectively inhibit the EMT phenotype in senescent HK‐2 cells, with the inhibitory effect being more pronounced for the GSK3β‐K15R point mutant plasmid. However, transfecting the GSK3β‐K15Q point mutant promotes EMT phenotypic characteristics. These findings suggest that deacetylation of GSK‐3β at K15 inhibits the EMT phenotype, whereas acetylation at this residue promotes EMT.

Western blot analysis of β‐catenin showed that transfection with the GSK3β‐K15R mutant significantly increased phosphorylated β‐catenin levels and decreased levels of active (non‐phosphorylated) β‐catenin compared to GSK3β‐WT transfection (Figure 5F). Immunofluorescence assays confirmed that nuclear accumulation of β‐catenin was markedly reduced in senescent HK‐2 cells transfected with the GSK3β‐K15R mutant (Figure 5G). Conversely, transfection with the GSK3β‐K15Q mutant decreased phosphorylated β‐catenin levels and increased active β‐catenin levels (Figure 5F). Correspondingly, immunofluorescence showed significantly elevated nuclear β‐catenin expression in GSK3β‐K15Q‐transfected cells (Figure 5G). Collectively, these results demonstrate that SIRT3 interacts with GSK‐3β and deacetylates it at K15. This deacetylation event promotes β‐catenin phosphorylation and degradation, thereby inhibiting Wnt/β‐catenin signaling and suppressing the EMT phenotype in senescent HK‐2 cells. As K183 has also been reported as an acetylation site on GSK3β, we generated a GSK3β‐K183R mutant plasmid using the same approach. However, cell experiments showed that mutation at K183 had no significant effect on the EMT phenotype in senescent HK‐2 cells, highlighting the specific importance of K15 deacetylation. (Figure S8, Supporting Information).

### 2.6 The Honokiol‐Loaded PEG‐PCL‐PEG Micelles Alleviate Renal Fibrosis in Aged UUO Mice

PEG‐PCL‐PEG micelles were prepared by the thermally induced self‐assembly of the triblock copolymer. Honokiol (HKL) was then loaded into these micelles (HKL‐micelles). As shown in Figure S9A (Supporting Information), HKL‐micelles could be lyophilized into a powder and readily reconstituted in aqueous solution, forming a stable and homogeneous dispersion. The amphiphilic PEG‐PCL‐PEG copolymer self‐assembles in aqueous solution due to the hydrophobic PCL segments forming the core and the hydrophilic PEG segments forming the corona, enabling efficient encapsulation of hydrophobic HKL, as shown in Figure S9A (Supporting Information). The drug loading ratio was 25.33 ± 2.78%, and the zeta potential was −0.35 mV. As expected, there was no significant initial burst release of HKL from the micelles, a characteristic two‐phase release pattern was observed. This involved a relatively fast release during the initial phase, followed by a prolonged and gradual release. After 125 h of incubation, the percentages of HKL released in solutions with pH of 7.4, 5.5, and plasma were ≈66.98%, 74.99%, and 64.89%, respectively. As expected, there was no significant initial burst release of HKL from the micelles, a characteristic two‐phase release pattern was observed. This involved a relatively fast release during the initial phase, followed by a prolonged and gradual release. After 125 h of incubation, the percentages of HKL released in solutions with pH of 7.4, 5.5, and plasma were ≈66.98%, 74.99%, and 64.89%, respectively (Figure S9B, Supporting Information).

Cytotoxicity assessment using the CCK‐8 assay showed that blank PEG‐PCL‐PEG micelles exhibited low toxicity to HK‐2 cells. Cell viability decreased gradually with increasing HKL concentration but remained above 80.56% even at the highest concentration tested (100 µm) after 24 h (Figure S9C, Supporting Information), indicating good biocompatibility and low cytotoxicity of the micellar carrier. The cell viability study implied that the HKL‐micelles were biocompatible, with low cytotoxicity. Throughout the entire observation period, animals treated with HKL‐micelles displayed normal energy levels, free movement, and shiny fur. Serum chemistry tests were conducted on the 7th day following the intravenous administration. No significant differences were observed in ALT, BUN, and CR levels between the micelle‐administered (HKL‐loaded, blank micelles) group and the control group (saline control) at a dose of 40 mg kg^−1^ (Figure S9D, Supporting Information). These findings indicate that the administration of HKL‐micelles did not adversely affect liver or kidney function. Pharmacokinetic studies revealed that the HKL‐micelles significantly improved the plasma profile of HKL compared to free HKL administered orally (Figure S9E, Supporting Information). Specifically, both the maximum plasma concentration (C_max_) and the area under the curve (AUC) were significantly higher for HKL‐micelles than for free HKL (Figure S9F, Supporting Information). Transmission electron microscopy (TEM) revealed that HKL‐micelles exhibited a uniform spherical morphology with an average diameter of 85.4 ± 9.5 nm (Figure S9G, Supporting Information).

**Figure 6:**
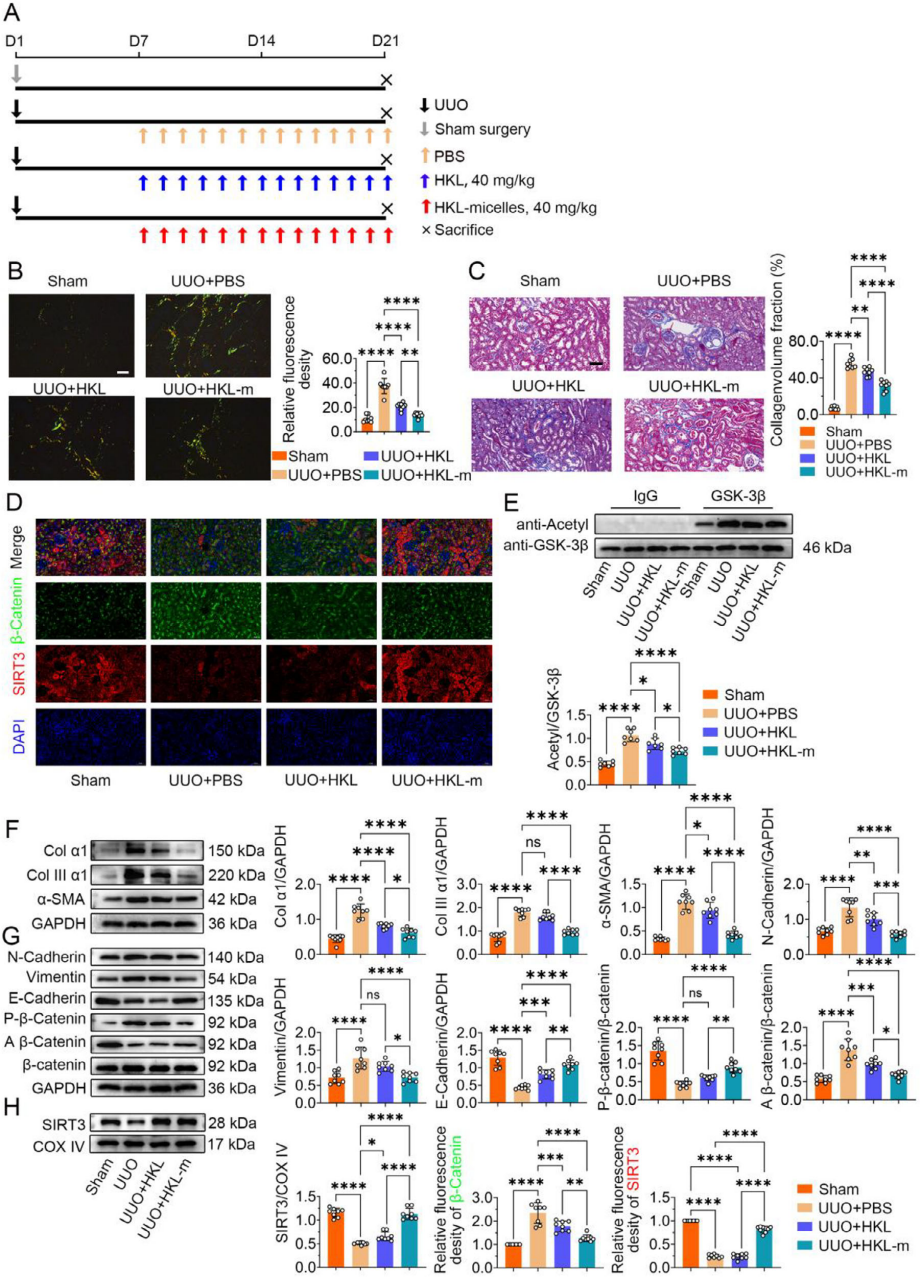
HKL‐micelles effectively attenuating renal fibrosis in aged UUO mice. A) The schematic diagram illustrates the sequential steps of the animal procedure. UUO, unilateral ureteral obstruction. B) Sirius red staining of kidney sections were examined in polarized light microscopy. Scale bar = 50 µm. C) Masson's trichrome staining of kidney sections. Masson fibrotic area percentage presented and analysis. Scale bar = 50 µm. D) Immunofluorescence staining showing SIRT3 and β‐Catenin protein expression. Scale bar = 50 µm. E) Western blots showing GSK3β protein acetylation level from kidney tissue. F) Western blots showing Col I α1, Col III α1, and α‐SMA protein expression. G) Western blots showing β‐Catenin, non‐phospho (Active) β‐Catenin‐S33/S37/T41, Phospho‐β‐Catenin‐S552, N‐cadherin, E‐Cadherin, and Vimentin protein expression in primary renal tubular epithelial cells. H) Western blots showing mitochondrial SIRT3 expression in primary renal tubular epithelial cells. *p < 0.05, **p < 0.01, ***p < 0.001, ****p < 0.0001, ns, no significance.

Next, HKL‐micelles were administered to treat renal fibrosis in aged UUO mice. The treatment protocol is illustrated in **Figure** **6**A. Sirius red and Masson's trichrome staining of kidney sections showed that both free HKL and HKL‐micelles treatment effectively attenuated the accumulation of renal interstitial fibrosis in aged UUO mice, with HKL‐micelles showing a more potent effect (Figure 6B,C). Western blot analysis further confirmed a significant reduction in the protein levels of fibrosis markers (collagen I α1, collagen III α1, and α‐SMA) in both treatment groups, with a more pronounced decrease in the HKL‐Micelles group (Figure 6F). Furthermore, the primary proximal tubular epithelial cells were isolated and western blot analysis revealed a significant decrease in active β‐catenin, N‐Cadherin, and Vimentin expression, while simultaneously upregulating E‐cadherin levels in the HKL and HKL‐micelles treatment groups (Figure 6G). In addition, HKL‐micelles treatment significantly increased SIRT3 protein expression in the kidneys of aged UUO mice and concomitantly decreased β‐catenin expression (Figure 6D). Furthermore, HKL‐micelles promoted the mitochondrial localization of SIRT3 in renal tubular epithelial cells (Figure 6H) and reduced the acetylation level of GSK‐3β (Figure 6E). In summary, we successfully synthesized HKL‐loaded PEG‐PCL‐PEG micelles. This nanocarrier markedly enhanced the solubility of hydrophobic HKL and demonstrated excellent stability and sustained release properties in vivo. Furthermore, the blank micellar carrier exhibited high biocompatibility. In aged UUO mice, treatment with HKL‐micelles significantly alleviated renal fibrosis, inhibited Wnt/β‐catenin signaling, and suppressed the EMT phenotype, demonstrating superior therapeutic efficacy compared to free HKL.

## 3 Discussion

The aging process is associated with a decline in renal function, along with macroscopic and microscopic histological changes, which significantly enhance the kidneys' susceptibility to injury and impair their recovery capacity. In this context, our study has uncovered some new and interesting phenomena and further elucidated the underlying molecular mechanisms. Our findings indicate that aging exacerbates UUO‐induced renal fibrosis, which is associated with a downregulated expression of SIRT3. Consistent with this role, knockout of SIRT3 exacerbated kidney fibrosis in young UUO mice, whereas supplementation of SIRT3 alleviated renal fibrosis in aged UUO mice. Mechanistically, the diminished expression of SIRT3 during the aging process is attributed to hypermethylation occurring within the promoter region of GSK3β, resulting in transcriptional repression. The deficiency of SIRT3 results in hyperacetylation of GSK3β at residue K15, exerting a negative regulatory effect on GSK3β and its substrates β‐catenin. By deacetylating and activating GSK3β, SIRT3 blocks the profibrotic Wnt/β‐catenin signaling pathway.

The expression of SIRT3 was found to decline progressively with advancing age, and the investigation into the methylation of SIRT3 from an aging perspective was first comprehensively documented by Silva et al.^[^ ^27^ ^]^ In that study, the MSP method was utilized to detect whole blood samples from individuals diagnosed with Alzheimer's disease (AD). However, their findings revealed no significant differences in SIRT3 methylation frequencies among young individuals, older adults, and AD patients, leading the investigators to postulate no discernible association between SIRT3 methylation and aging or AD. The composition of whole blood is highly intricate, and many influencing factors may cause negative results of whole blood testing. Unfortunately, the investigators did not pursue further studies on this aspect. Consistent with reports of age‐related decline in other tissues, we also observed decreased SIRT3 expression in kidney tissue from aged mice and elderly individuals. Furthermore, H₂O₂‐induced cellular senescence in HK‐2 cells was associated with a marked downregulation of SIRT3 expression. As predicted, SIRT3 gene promoter regions contain characteristic CpG islands within the 1008–1610 and 1764–2195 regions, and we also confirmed a higher level of SIRT3 promoter methylation in tissues collected from elderly individuals and senescent HK‐2 cells through MSP analysis. Importantly, treatment with 5aza, a DNA demethylating agent, could significantly restored SIRT3 expression by inhibiting DNA methylase activity in senescent HK‐2 cells. Our study provides preliminary evidence of novel findings on the DNA methylation mechanism responsible for downregulating SIRT3 expression during renal aging. Therefore, DNA demethylating agents, by restoring SIRT3 expression, represent a potential therapeutic strategy for mitigating renal aging. Nevertheless, additional in vivo studies are required to substantiate this hypothesis.

To date, available evidence suggests a protective role of SIRT3 against fibrosis primarily in cardiac tissues,^[^ ^28^ ^]^ liver,^[^ ^29^ ^]^ and lung,^[^ ^30^ ^]^ with limited data available for renal fibrosis.^[^ ^17^, ^31^ ^]^ In our study, phenotypic experiments demonstrated that age‐related reduction in SIRT3 expression exacerbates renal fibrosis in UUO model mice. Consequently, we propose that age‐related SIRT3 downregulation is a pivotal factor promoting UUO‐induced renal fibrosis. We further generated a UUO model by employing young conditional SIRT3 knockout mice, in which SIRT3 expression was specifically depleted in renal tubular epithelial cells. It was noted that mice lacking SIRT3 exhibited significant kidney fibrosis when compared to their age‐matched WT counterparts. Additionally, SIRT3 supplementation attenuated UUO‐induced fibrotic alterations in aged mice. Consistent with this, overexpression of SIRT3 suppresses TGFβ‐associated fibrogenic phenotype in senescent HK‐2 cells. SIRT3 supplementation attenuated UUO‐induced fibrotic alterations in aged mice.

Integrative analysis of RNA‐seq and IP‐MS data revealed that the molecular mechanism underpinning our findings primarily involves the Wnt/β‐catenin signaling pathway and GSK3β. For in vitro validation, we employed LiCl‐treated senescent HK‐2 cells as a positive control (LiCl activates Wnt signaling). LiCl treatment significantly activated the Wnt/β‐catenin pathway in senescent HK‐2 cells, manifested by increased levels of active β‐catenin and upregulated expression of c‐MYC and cyclin D1. SIRT3 overexpression elevated phosphorylated β‐catenin levels, while reducing c‐MYC and cyclin D1 expression and TCF/LEF transcriptional activity. Additionally, SIRT3 overexpression suppressed the LiCl‐induced upregulation of vimentin and N‐cadherin and restored E‐cadherin expression. These findings confirm that SIRT3 inhibits EMT by negatively regulating the Wnt/β‐catenin signaling pathway. The Wnt/β‐catenin is typically quiescent in healthy adult kidneys, yet it undergoes reactivation in various forms of CKD and assumes a pivotal role in driving disease progression. Our findings complement existing research by highlighting the significant contribution of Wnt/β‐catenin signaling to age‐associated fibrosis.

GSK3β, a serine/threonine protein kinase constantly active and widely conserved, plays a crucial role in regulating the Wnt/β‐catenin signaling pathway.^[^ ^32^ ^]^ In the context of GSK3β activity regulation, the mechanism most commonly investigated is inhibitory serine‐phosphorylation. However, a recent study has highlighted the occurrence of acetylation on GSK3β in cardiac hypertrophy.^[^ ^33^ ^]^ Our data reveal that SIRT3 directly binds to and deacetylates GSK3β, enhancing its kinase activity and consequently inhibiting β‐catenin‐mediated expression of fibrotic genes. Furthermore, we identified acetylation at GSK3β lysine 15 (K15) as a critical regulatory site. SIRT3 is capable of interacting with GSK‐3β and performing its deacetylase activity to facilitate the deacetylation of the 15th amino acid residue in GSK‐3β. Deacetylation of K15 inhibits Wnt signal transduction and suppresses the EMT phenotype in senescent cells. In line with our findings, a previous investigation has indicated that the involvement of residue K15 in facilitating the translocation process of GSK3β into mitochondria was observed.^[^ ^34^ ^]^ Collectively, these reports and our data suggest that the mitochondrial deacetylase SIRT3 specifically activates GSK3β via deacetylation. Our results indicate that SIRT3‐dependent deacetylation of GSK3β‐K15 is crucial for regulating the Wnt/β‐catenin signaling pathway. Recently, Xin et al.^[^ ^21^ ^]^ made a significant discovery regarding the close association between GSK3β activity and its acetylation status regulated by SIRT3 during oocyte meiosis. Our study significantly contributes to the understanding of age‐related fibrosis by elucidating the substantial role of Wnt/β‐catenin signaling. Honokiol (HKL) has demonstrated anti‐inflammatory and anti‐fibrotic effects in studies involving neutrophils and hepatic fibrosis. Chiang et al.^[^ ^35^ ^]^ provided the first evidence supporting HKL's potential as a therapeutic agent for renal fibrosis. To address HKL's hydrophobicity, we engineered self‐assembled PEG‐PCL‐PEG micelles for its encapsulation. These amphiphilic block copolymers self‐assemble into micelles with a hydrophobic core (PCL blocks) encapsulating HKL and a hydrophilic corona (PEG blocks) providing colloidal stability and dispersion.^[^ ^24^ ^]^ This micellar formulation effectively overcomes HKL's poor aqueous solubility and meets the requirements for intravenous administration. The HKL‐loaded micelles exhibited uniform nanospherical morphology and enabled sustained release of HKL. This sustained‐release profile allows for reduced dosing frequency while maintaining stable plasma drug concentrations, potentially enhancing anti‐fibrotic efficacy.

In summary, our study proposes a mechanism whereby SIRT3 indirectly inhibits Wnt/β‐catenin signaling activation by deacetylating GSK3β, thereby regulating age‐related fibrotic tissue remodeling. However, several key questions remain: 1) The structural basis and functional consequences of K15 (and other potential sites) deacetylation on GSK3β kinase activity require elucidation. 2) Whether additional deacetylation sites on GSK3β exist and contribute to regulation needs exploration. 3) Potential crosstalk between acetylation, phosphorylation, and other post‐translational modifications of GSK3β warrants investigation. 4) While HKL‐micelles show therapeutic promise for renal fibrosis, comprehensive material characterization and rigorous in vivo efficacy/safety assessment in relevant animal models are essential next steps.

## 4 Conclusion 

This study delineates the decline of SIRT3 during aging and establishes its significance as a key biomarker and mediator of age‐related renal fibrosis. Upregulating SIRT3 alleviates renal fibrosis progression by modulating GSK3β acetylation (specifically deacetylating K15), thereby indirectly inhibiting Wnt/β‐catenin signaling. Furthermore, HKL‐loaded micelles significantly attenuated renal fibrosis in aged UUO mice. Our findings unveil novel therapeutic strategies for treating renal fibrosis in the elderly population and provide a foundation for developing innovative drugs and combination therapies.

## 5 Experimental Section

### Reagents and Antibodies

PEG (# 1546569, Sigma‐Aldrich, CAS. 25322‐68‐3), ε‐caprolactone (# 8.02801, Sigma–Aldrich, CAS.502‐44‐3), stannous 2‐ethyl‐hexanoate (Sn(Oct)2) (# S3252, Sigma–Aldrich CAS. 301‐10‐0), H_2_O_2_ (# 216763, Sigma–Aldrich), TGF‐β1 (# RP01458, ABclonal), Lithium chloride hydrate (LiCl, # HY‐W094474, MedChemExpress), 5‐Aza‐2’‐deoxycytidine (5‐aza‐dC, # 189825, Sigma–Aldrich), GNE‐272 (# HY‐100726, MedChemExpress), Honokiol (HKL, # 42612, Sigma–Aldrich, CAS. 35354‐74‐6) were procured from commercial sources. Anti‐DNMT1 (# A22455), anti‐DNMT3A (# A19659), anti‐DNMT3B (# A22658), anti‐Collagen I α1 (# A1352), anti‐E‐Cadherin (# A20798), anti‐Collagen III α1(# A0817), anti‐β‐Catenin (# A19657), anti‐Vimentin (# A19607), anti‐Phospho‐β‐Catenin‐S552 (# AP1315), anti‐N‐Cadherin (# A19083), anti‐α‐Smooth Muscle Actin (# A17910), anti‐Non‐phospho (Active) β‐Catenin‐S33/S37/T41 (# A22180) were purchased from ABclonal Technology Co., Ltd. . Anti‐Acetyllysine (# PTM‐105RM) was purchased from PTM BIO Co., Ltd. DYKDDDDK tag Monoclonal antibody (Binds to FLAG® tag epitope) was purchased from Proteintech Co., Ltd. Anti‐SIRT3 (# ab246522) was obtained from Abcam Co., Ltd. Species‐specific secondary antibodies (anti‐mouse, anti‐rabbit, etc.) and DAPI were sourced from ABclonal. Technology Co., Ltd. Fluorescent secondary antibodies (ab150076, ab150116) were obtained from Abcam Co., Ltd.

### Clinical Specimens

The kidney tissues were collected from patients who underwent radical nephroureterectomy for renal cancer (older adults, >60 years, *n* = 6; younger adults, <40 years, *n* = 6). Normal renal tissue (adjacent tissues far from the tumor) was used in this study. Detailed case information is presented in Table S1 (Supporting Information). This study, conducted at Tongji Hospital, Tongji Medical College, Huazhong University of Science and Technology, was approved by the Institutional Review Board (Approval No. TJ‐C20200155) on January 15, 2020, and adhered to the principles of the Declaration of Helsinki (1964). Informed consent was obtained from all participants.

### Animal Study

The Basel Declaration was strictly adhered to during all animal experiments, and all procedures were approved by the Animal Ethics Committee of Tongji Medical College (Approval No. TJ‐A20211214) on December 14, 2021, and strictly followed the Basel Declaration. The SIRT3^flox/flox^ mice (C57BL/6 background) were acquired through Shulaibao Biotechnology Co., Ltd. (Wuhan, China; Project No. SLB426). These mice were then bred with Cdh16‐Cre mice to generate a specific knockout of SIRT3 in renal tubules. The flox modification scheme of the SIRT3 gene and the mating breeding protocol for the mice were illustrated in Figure S1 (Supporting Information). Genotyping was performed by PCR (Supporting information_Genotype identification report). For the UUO model, the mice were anesthetized, and a lateral incision was made on the abdomen. Subsequently, meticulous dissection and ligation of the left ureter with a 4.0 silk suture were performed. The surgical procedure for mice in the Sham group mirrored that of the experimental group, except for the omission of ureteral ligation. Adeno‐associated virus type 9 (AAV9)‐SIRT3 (AAV9‐CMV‐Mouse_SIRT3‐T2A‐eGFP‐WPRE) were generated by Genomedtech (Shanghai, China, Project No. GM‐AA‐134550). Intravenous injection of AAV9‐SIRT3 and AAV‐eGFP into the tail vein was administered to mice (1.0 µL, 1.0 × 10^12^ vg µL^−1^). After 3 weeks, the efficiency of viral infection was assessed through immunofluorescence staining for eGFP (Figure S4, Supporting Information).

To assess the impact of age on renal fibrosis, a total of four distinct groups (*n* = 5–8 per group) were formed for the experimental mice: Sham–Young group, Sham‐Old group, UUO‐Young group, and UUO‐Old group. Female/male C57BL/6 mice (young, aged 6–8 weeks; old, aged 22–24 months) were used, purchased from Hubei BIONT (Wuhan) Biotechnology Co., Ltd. Further exploration of the role of SIRT3 in renal fibrosis, in the second phase of the in vivo study, a group of young mice (aged 6–8 weeks) was divided into four categories (*n* = 5–8 per group): Sham‐WT, Sham‐SIRT3^‐/‐^, UUO‐WT, and UUO‐SIRT3^‐/‐^. In the third phase of the in vivo experiment, a group of aged mice (22–24 months old) were divided into four groups, with each group consisting of 5–8 mice: Sham‐AAV9‐empty group, Sham‐AAV9‐SIRT3 group, UUO‐AAV9‐empty group, and UUO‐AAV9‐SIRT3 group. The schematic diagram of the experimental procedure is presented in Figure 3. After the animals were sacrificed, the kidney tissues were obtained for further analysis. For in vivo anti‐fibrosis studies, in the fourth phase of the animal experiment, a cohort of aged mice (22–24 months old) were categorized into four distinct groups, each consisting of 5–8 mice: Sham group, UUO+vihicle group, UUO+HKL group, and UUO+HKL‐micelles group. The mice were subjected to unilateral ureteral obstruction (UUO) as a pretreatment. Drug treatments commenced 1 week after UUO surgery, when fibrosis was established. Subsequently, drug treatments were administered daily at an HKL‐equivalent dose of 40 mg kg^−1^, either as free drug (free HKL) or encapsulated in micelles (HKL‐micelles). Treatment continued for two weeks. At the end of the treatment period, animals were euthanized, and renal tissues were harvested for further analysis.

### Cell Culture, Transfection, and Treatment

The HEK‐293T cells (# SCSP‐5209) and HK‐2 cell line (# SCSP‐511) were obtained from the National Collection of Authenticated Cell Cultures. For the HK‐2 cells, a medium consisting of Dulbecco's modified Eagle's medium (DMEM)/F12 supplemented with 10% fetal bovine serum (FBS) was utilized, while DMEM supplemented with 10% FBS was employed to maintain the HEK‐293T cells. The mouse primary tubular epithelial cells (mPTEC) were isolated and subcultured in DMEM/F12 supplemented with 10% FBS.

The overexpression plasmids (PCDH‐CMV‐GSK3β‐3×Flag‐EF1a‐copGFP‐T2A‐Puro, pCDH–CMV‐SIRT3‐EF1‐copGFP‐T2A‐Puro, PCDH‐CMV‐GSK3β‐Mut (K15R, K15Q)‐3×Flag‐EF1a‐copGFP‐TA‐Puro) and their corresponding empty vectors were obtained from GENERAL BIOL Co., Ltd. (Hefei, China, # G0244862‐1, # G0280258‐3). The HK‐2 were transfected with the overexpression plasmids or the empty vector alone using Lipofectamine™ 3000 reagent (Thermo Fisher Scientific, USA).

The HK‐2 cells underwent a 2‐day treatment with TGF‐β1 (10 ng mL^−1^) to induce epithelial‐mesenchymal transition (EMT). In this study, H₂O₂ was utilized to induce senescence in HK‐2 cells, and dose of 200 µm for 48 h was selected as the optimal condition to induce senescence, and senescence induction was confirmed by analysis of p16 and p21 expression, and by senescence‐associated β‐galactosidase (SA‐β‐Gal) staining (Figure S2, Supporting Information).

### Histology, Immunohistochemistry, and Immunofluorescence Staining

H&E staining was used to assess renal morphology. Collagen deposition and fibrosis were evaluated using Masson's trichrome or Sirius red staining. For immunohistochemical analysis purposes, the sections were incubated with primary antibody against SIRT3 (# ab246522, Abcam, USA) overnight at 4 °C. Afterward, sections were incubated with secondary antibodies and were subsequently counterstained with hematoxylin. Images were acquired using a Leica microscope (Germany).

To perform immunofluorescence staining on tissue samples, paraffin‐embedded tissue sections were used. For the staining of cells using immunofluorescence, cells were cultured on climbing slides and fixed. Following sample blocking with a 5% bovine serum albumin (BSA), primary antibodies (SIRT3 1:100, α‐SMA 1:100, β‐catenin 1:100) were applied and incubated. The climbing pieces or sections were incubated with fluorophore‐conjugated secondary antibodies. After staining with DAPI and sealing the sections, fluorescence microscopy from Olympus in Japan was used for image visualization.

### Western Blot Analysis

The RIPA lysis buffer (BL504A, Biosharp, China) was utilized to extract the total protein. The BCA assay (BL521A, Biosharp, China) was employed to determine the concentration of solubilized proteins. Protein samples (30 µg) or prestained protein markers (catalog # 26616, 10–180 kDa; # 26620, 10–250 kDa, Thermo Fisher Scientific) were separated by sodium dodecyl sulfate‐polyacrylamide gel electrophoresis (SDS‐PAGE) using 6%, 10%, or 15% gels, followed by transferred to polyvinylidene difluoride (PVDF) membranes (# ISEQ00010, Millipore). For normalization purposes, GAPDH was used as the internal housekeeping gene.

### Co‑Immunoprecipitation (Co‑IP)

The Abbkine Universal IP/Co‐IP Kit (# KTI1010‐CN, Abbkine, China) was utilized to conduct IP/Co‐IP experiments. In brief, cells were lysed in a non‐denaturing lysis buffer. The antibodies against SIRT3 or Flag were used for immunoprecipitation overnight. Protein A/G magnetic beads were used to capture the proteins bound by the antibodies. Whole‐cell lysates served as an input control. To assess the acetylation level of GSK3β, cells were lysed in a non‐denaturing lysis buffer. The Flag antibody was used for immunoprecipitation overnight at 4 °C. The immunoprecipitates were subjected to three washes followed by boiled in 1× SDS loading buffer for subsequent analysis using immunoblotting.

### Quantitative Real‐Time Reverse Transcription Chain Reaction (qRT‐PCR)

Total RNA was isolated from freshly obtained kidney tissues or cells utilizing the FastPure Cell/Tissue Total RNA Isolation Kit (# RC101‐01, Vazyme Biotech, China). According to the manufacturer's instructions, the first‐strand cDNA was synthesized using the HiScript III RT SuperMix Kit (R323‐01, Vazyme Biotech, China). Amplification was performed on the QuanStudio 6 fluorescence quantitative PCR detection system (ThermoFish Scientific). The SYBR qPCR Master Mix (# Q712‐02, Vazyme Biotech, China) was utilized for the qPCR assay. Normalization of mRNA levels to GAPDH and data analysis were conducted using the 2 ^–ΔΔCT^ method. The primer sequences used in this study are shown in Table S2 (Supporting Information).

### Methylation‐Specific PCR (MSP)

CpG islands in the human SIRT3 promoter region was assessed using MetPrimer software (http://www.urogene.org/). Genomic DNA was extracted using the FastPure Isolation Mini Kit (# DC102‐01, Vazyme, China) and subjected to bisulfite modification with the Methylation Bisulfite Kit (# EM101‐01, Vazyme, China), following the provided instructions. Methylation‐specific primers (for methylated DNA) and unmethylation‐specific primers were designed along with 2×EpiArt® HS Taq Master Mix Kit (EM201‐01, Vazyme Biotech, China) for PCR amplification under predetermined conditions. The PCR products obtained were observed using a gel made of agarose with a concentration of 1.5% and analyzed using ImageJ software for densitometry analysis. Table S3 (Supporting Information) contains the primer sequences.

### TOPFlash/FOPFlash

The TCF/LEF transcriptional activity was evaluated by determining the ratio between TOP and FOP. The HK‐2 cells were subjected to transfection with the TOP‐Flash/FOP‐Flash luciferase reporter gene and Renilla luciferase transfection control reporter gene using Lipofectamine™ 3000. Following a 24‐h incubation period, TOP‐Flash's luciferase activity was quantified using the luciferase reporter assay system (Promega, Madison, WI, USA).

### RNA Sequencing

Kidney samples were obtained from two groups: young mice subjected to UUO (young_UUO) and old mice subjected to UUO (old_UUO). Each group contained 3–5 mice. The mRNA expression levels in these samples were analyzed by isolating mRNA sing Dynabeads Oligo (DT) from Thermo Fisher, CA, USA. Subsequently, cDNA was generated, and paired‐end sequencing (PE150) was then performed using the Illumina Novaseq™ 6000 instrument provided by LC‐Bio Technology CO., Ltd., Hangzhou, China. Differential gene expression analysis was performed using DESeq2 software, with a significance threshold set at *p* < 0.05 and an absolute fold change>1. More comprehensive document of methodologies for RNA extraction, library construction, and sequencing data analysis were recorded in the Supporting information_RNA extraction library construction and sequencing.

### Immunoprecipitation Followed by Mass Spectrometry (IP‐MS)

To establish a stable HK‐2 cell line expressing SIRT3, HEK‐293T cells were transfected with pCDH–CMV‐SIRT3‐EF1‐copGFP‐T2A‐Puro or an empty vector, along with packaging vectors, using Lipofectamine™ 3000. Lentiviral supernatants were harvested following a 72‐h incubation period to infect HK‐2 cells. Subsequently, puromycin dihydrochloride (MCE, HY‐B1743A) was employed at a concentration of 1 µg mL^−1^ to select a polyclonal HK‐2 cell line exhibiting stable expression of SIRT3. For the IP‐MS analysis, stable HK‐2 cells that expressed SIRT3 were obtained and protein extraction was conducted using a previously described method, as described in Section “Western Blot Analysis.” 50 µl of protein A/G magnetic beads were introduced into the solution containing the protein‐antibody mixture. The IP‐MS procedure and subsequent data analysis were entrusted to SpecAlly Life Technology Co., Ltd. (Project No. 23053001). A timsTOF Ultra Mass spectrometer (BRUKER, USA) with an UltiMate 3000 RSLCnano Liquid Chromatograph (ThermoFisher Scientific, USA) was employed to preprocess and analyze the samples. The initial mass spectrometric data underwent bioinformatics and statistical analysis using MaxQuant_V2.2.0.0 software, which utilized the Andromeda database search algorithm for querying against UniProt's Human Proteome Reference Database (https://www.uniprot.org/).

### Preparation and Characterization of Honokiol‐Loaded PEG‐PCL‐PEG Micelles

The PEG‐PCL‐PEG copolymers were synthesized following the previously reported method^[^ ^24^ ^]^ and detailed preparation process and characteristic detection data are shown in the Supporting information_Synthesis and characterization of the PEG‐PCL‐PEG copolymers. The HKL‐loaded micelles were prepared via the nanoprecipitation method employing acetone. Briefly, PEG‐PCL‐PEG copolymer (20 mg) and HKL (5 mg) were dissolved in 2 mL of acetone. The solution was injected drop‐wise through a syringe (G = 22) into 25 mL of distilled water under certain mixing rates and stirred magnetically at room temperature until complete evaporation of the organic solvent, which produced the amphiphilic copolymers to self‐associate to form the micelles. The resulting aqueous solution was filtered through a 0.45 µm filter membrane to remove the unloaded HKL. The resulting micelles were separated by centrifuging at 20 000 g for 20 min and freeze‐dried under a pressure of 14 Pa at −80 °C in order to remove all the residual solvents and to produce the final dried form.

The morphology and particle size of micelles were determined by transmission electron microscope (Hitachi TEM system, Japan). Besides, in order to confirm the existence of HKL‐loaded micelles in vivo, the kidney specimens were immersed in electron microscope fixative and also observed by TEM.

To determine the drug‐loading ratio, 1mg of freeze‐dried nano micelles was dissolved in 1 mL of chloroform and the concentration of the drug was analyzed using high‐performance liquid chromatography (HPLC, SHIMADZU LC‐40D, Japan). HPLC analysis was performed using an Alliance 2695 system (Waters Corp., Milford, MA, USA) equipped with an automated sample manager maintained at 10 °C and a SunFire™ C18 column (150 mm × 4.6 mm, 5 µm) maintained at 28 ± 0.5 °C. Isocratic elution was carried out with a mobile phase of acetonitrile/water (60:40, v/v) at a flow rate of 1.0 mL min^−1^. Calibration curves were established over the concentration range of 0.1–50 µg mL^−1^ (R^2^ > 0.999). Drug loading ratio was determined as:

(1) $$drug\;loading\;ratio=\frac{\mathrm{weight}\;\mathrm{drug}\;\mathrm{in}\;\mathrm{micelles}}{\mathrm{weight}\;\mathrm{micelles}}$$

The drug release study was performed on HKL‐micelles in neutral (pH 7.4) and acidified PBS solution (pH 5.5) as well as the freshly prepared plasma withdrawn from c57BL/6 mice. Briefly, 5 mg of freeze‐dried drug‐loaded carriers were dispersed in 2 mL PBS and incubated at 37 °C. Then, at predetermined time intervals, 200 µL of the dialysate was taken out and replaced by 200 µL fresh PBS. The HKL concentration in the dialysate was measured using HPLC. As control, the release of free HKL was also studied in PBS.

For the biocompatibility evaluation of the material, CCK‐8 test was used in vitro (HK‐2 cells). In vivo biocompatibility was assessed by measuring serum markers of liver function (ALT, AST), renal function (BUN, creatinine), and cardiac injury (CK‐MB) in treated animals.

An oral pharmacokinetic study of both the free drug and nano‐micelle formulation of HKL was performed. Mice were randomly allocated into two groups, with each group receiving either the free drug or the nano‐micelle formulation. Blood samples were collected via cardiac puncture at 0, 0.5, 1, 2, 4, 8, 12, 18, and 24 h post‐administration via cardiac puncture. A separate cohort of mice (n = 3–4) was used for each time point. The drug was extracted from plasma samples using protein precipitation, and the magnolol concentration in the samples was quantified through HPLC. Key pharmacokinetic parameters, including the area under the curve (AUC) and the maximum plasma concentration (C_max_), were subsequently evaluated.

### Statistical Analysis

Data are presented as mean ± standard deviation (SD), and SPSS version 19.0 was used for statistical analysis. Normality plots and Levene variance homogeneity tests were employed to evaluate the normal distribution and equality of variances in the data. Two independent‐sample *t*‐tests were utilized to compare data groups with similar variances. If variances were unequal, Welch's *t*‐test was applied. For comparisons among three or more groups with normally distributed data, one‐way or two‐way analysis of variance (ANOVA) was performed, followed by appropriate post‐hoc tests (Tukey's or Sidak's test). For data that did not meet the assumptions of normality, nonparametric tests were used: Mann–Whitney U test for two‐group comparisons and Kruskal–Wallis test followed by Dunn's post‐hoc test for multiple‐group comparisons. The threshold for statistical significance was established at *p* < 0.05.

## Conflict of Interest

The authors declare no conflict of interest.

## Author Contributions

All authors played a significant role in conceptualizing and designing the study. J.W. was responsible for the preparation and execution of this research. X.R., Z.H.G., and Y.Q.T. were involved in gathering patient information. X.L., H.L., Z.L.Q., and Y.S.Y. conducted statistical calculations for this study. X.Y.Z., G.C., and M.L.W. were responsible for the research design. All authors thoroughly examined the manuscript and actively participated in its extensive revision before submission. All authors have carefully perused and approved the final version of the manuscript.

## Compliance with Ethics Guidelines

The research conducted at Tongji Hospital, Tongji Medical College, and Huazhong University of Science and Technology received approval on January 15, 2020, with the No TJ‐C20200155. Adhering to the 1964 Declaration of Helsinki, this study obtained informed consent from all participating patients. Moreover, the Basel Declaration was strictly adhered to during all animal experiments, and approval was received from the Animal Ethics Committee of Tongji Medical College on December 14, 2021, under the No TJ‐A20211214.

## Supporting information



Supporting Information

## Data Availability

The data that support the findings of this study are available from the corresponding author upon reasonable request.
