# Supplementary material for: Restoration of SIRT3 Expression in Aged Mice Alleviates UUO‐Induced Renal Fibrosis by Reducing GSK‐3β Hyperacetylation
Source: Adv Sci (Weinh). 2025 Jul 23;12(39):e17248. doi: 10.1002/advs.202417248 (PMC12533413; doi:10.1002/advs.202417248)

ADVANCED SCIENCE
Open Access

Supporting Information

for *Adv. Sci.*, DOI 10.1002/advs.202417248

Restoration of SIRT3 Expression in Aged Mice Alleviates UUO-Induced Renal Fibrosis by Reducing GSK-3β Hyperacetylation

*Jing Wang, Xiang Ren, Huan Lu, Zihao Guo, Xing Li, Yiqun Tian, Yisheng Yin, Zhenliang Qin, Kun Yun, Minglong Wu, Gang Chen and Xiaoyong Zeng**

Supporting Information - Figures

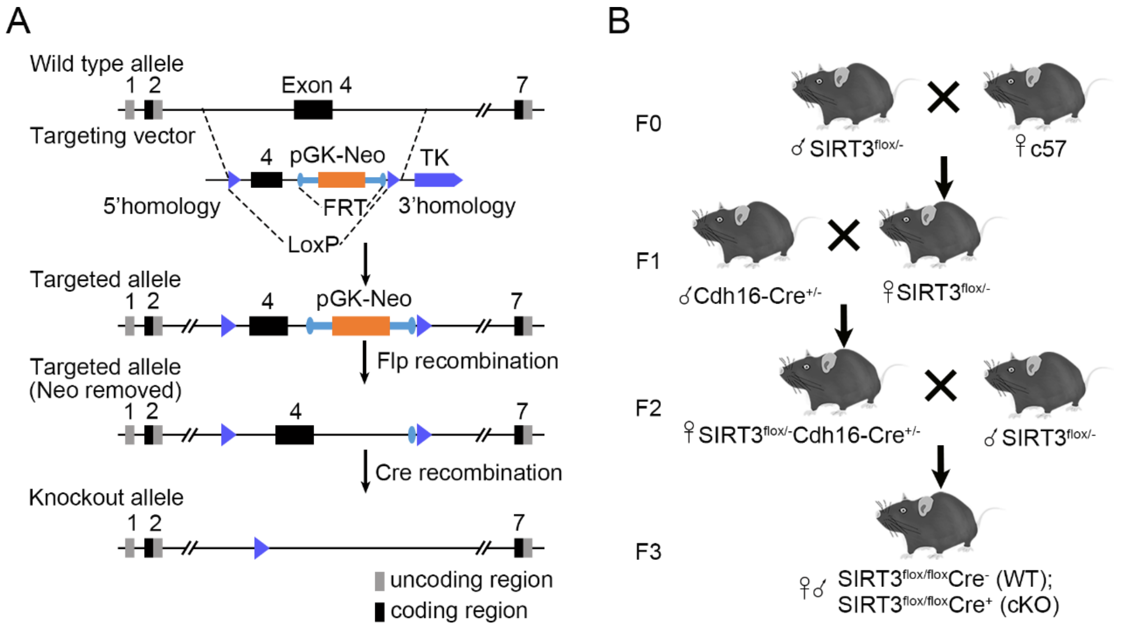

Figure S1.

Schematic illustration of the generation of renal tubular specific SIRT3 knockout mice. A) The targeting vector comprised a 2.6 kilobase (kb) 5' homology arm, a 0.7 kb flox region, PGK-Neo-polyA, a 3.2-kb 3' homology arm, and an MC1-TK-polyA negative screening marker. The linearized vector was transfected into JM8A3 embryonic stem cells by electroporation. Reproductive mice with chimeric characteristics were obtained by injecting expanded embryonic stem cell clones, identified as positive through long PCR, into the blastocysts of C57BL/6J mice. B) The chimeric mice were bred with Flp mice to acquire heterozygous mice that lacked the Neo gene. The flox mice were then bred with Cdh16-Cre mice to generate a specific knockout of SIRT3 in renal tubules. Genotyping was determined through PCR (Supporting Information - Genotype identification report).

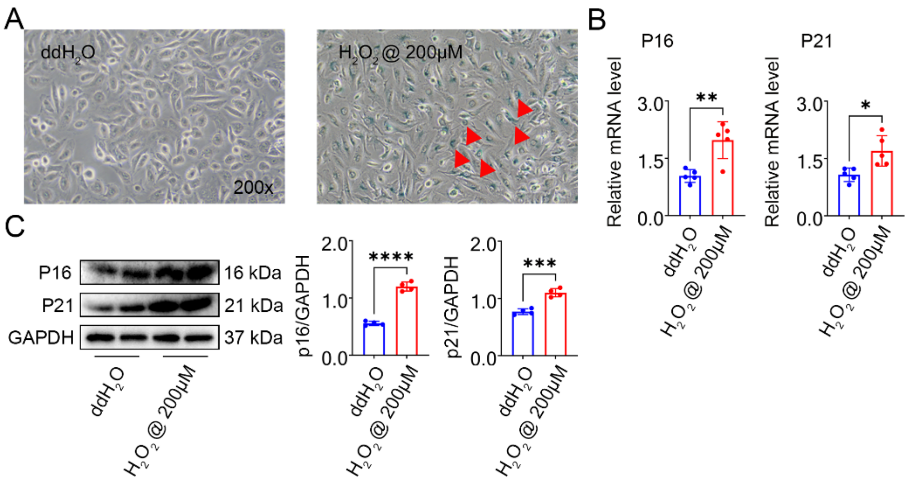

Figure S2.

H_2O_2 treatment induces cellular senescence in HK-2 cells. A) Morphological characteristics and increased SA-β-gal activity of HK-2 cells treated with 200 μM H_2O_2. 200× magnification. Red arrows show senescent cells with blue-stained nuclei. B) qRT-PCR showed p16 and p21 mRNA expression. Primers for senescence-associated mRNA: CDKN1A (p21) forward: *GACACCACTGGAGGGTGACT*; reverse: *CAGGTCCACATGGTCTTCCT*. CDKN2A (p16) forward: *CCAACGCACCGAATAGTTACG*; reverse: *GCGCTGCCCATC ATCATG*. C) Western blots showing p16 and p21 protein expression. *$p < 0.05$, **$p < 0.01$, ***$p < 0.001$, ****$p < 0.0001$.

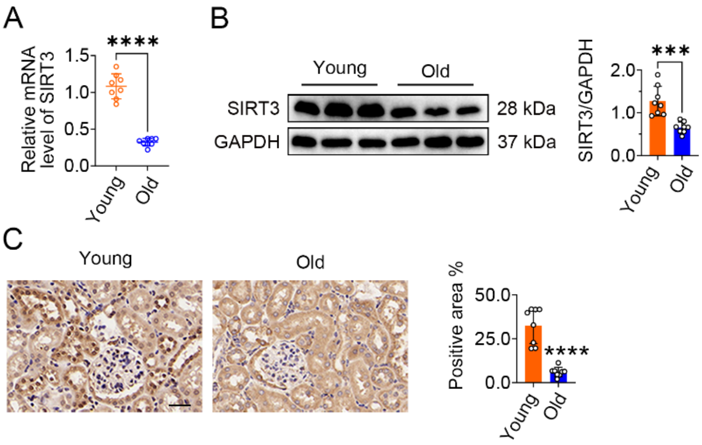

Figure S3.

SIRT3 exhibits downregulation in the aging kidney. A) qRT-PCR showing SIRT3 mRNA expression in mice. B) Western blots showing SIRT3 protein expression level. C) Immunohistochemistry signals of SIRT3 in mice kidney tissue. Scale bar, 50 μm. ***$p < 0.001$, ****$p < 0.0001$.

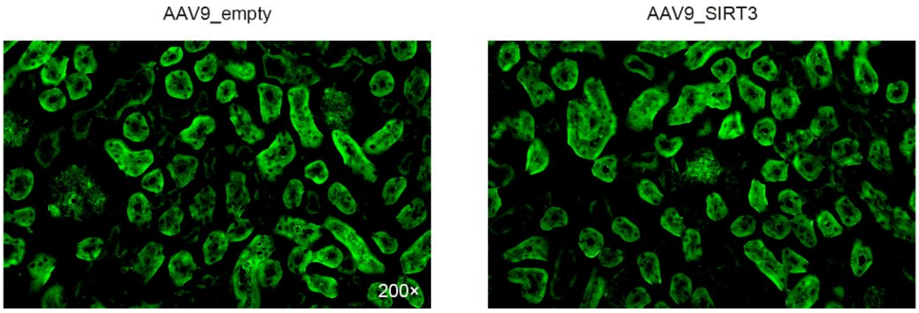

Figure S4.

Efficiency of viral infection was assessed through immunofluorescence staining for eGFP. 200× magnification.

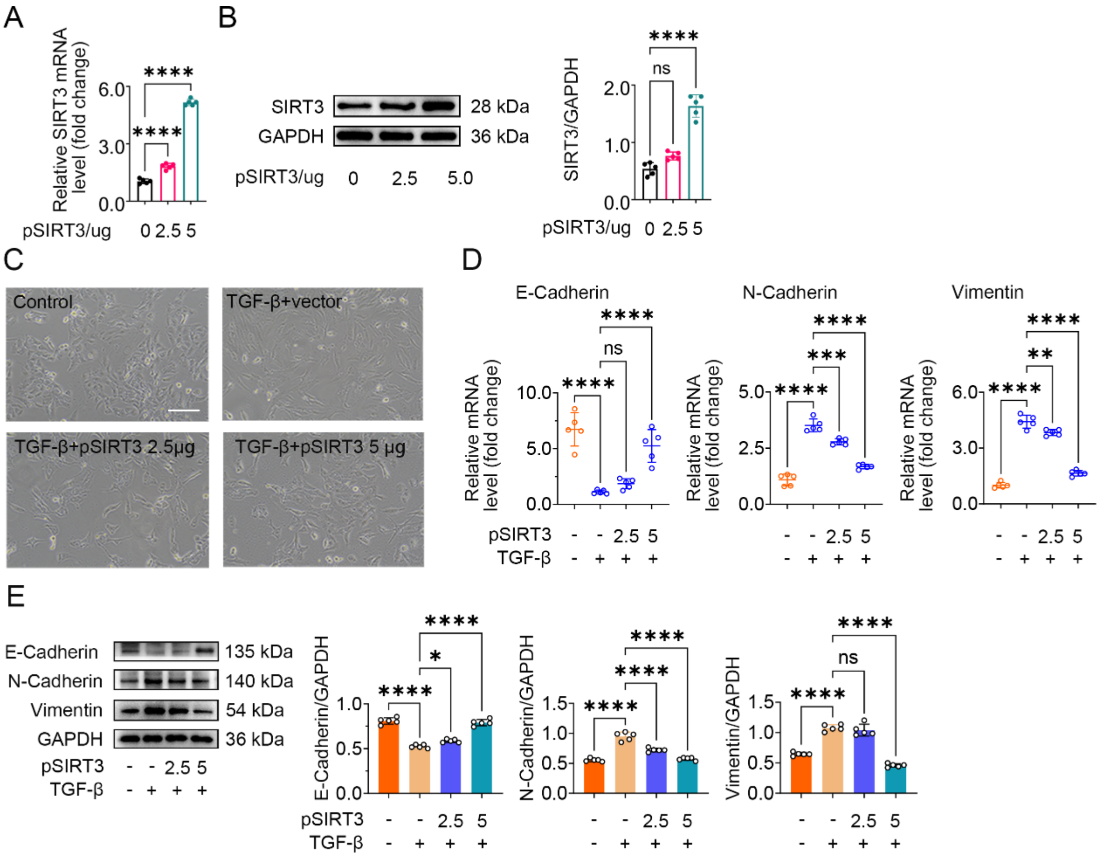

Figure S5.

SIRT3 overexpression suppresses TGFβ-associated fibrogenic features in senescent HK2 cells. A, B) The overexpression efficiency of the SIRT3-plasmid was validated through qRT-PCR and western blot analyses, respectively. C) Phase-contrast image of senescent HK-2 cells. Scale bar, 50 µm. D, E) qRT-PCR and western blots showing N-cadherin and Vimentin expression. *$p < 0.05$, **$p < 0.01$, ***$p < 0.001$, ****$p < 0.0001$.

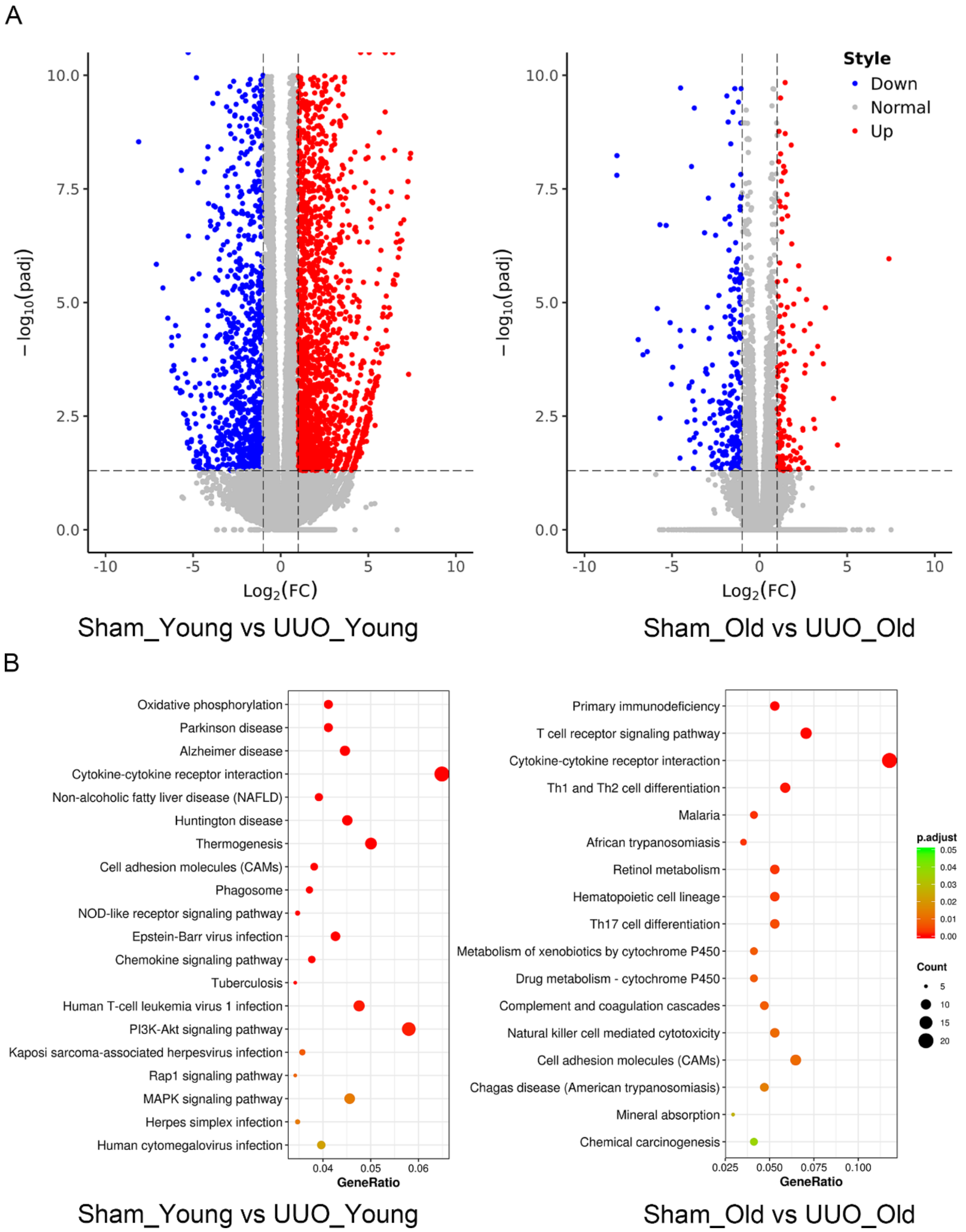

Figure S6.

RNA-seq data analysis. A) Volcano plot shows differentially expressed genes. Sham_Young vs UUO_Young, Sham_Old and UUO_Old. B) Conducting KEGG pathway enrichment analysis on the genes that exhibit differential expression. The top 20 enriched pathways were shown.

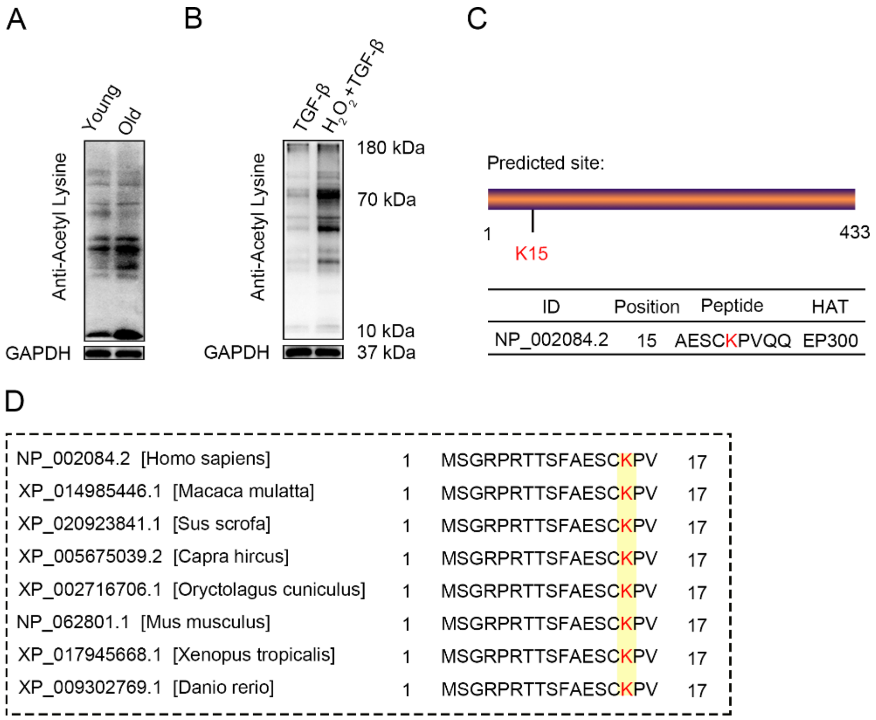

Figure S7.

GSK-3β acetylation site prediction and protein species conservation analysis. A, B) Western blots showing total protein acetylation level from kidney tissue and senescent HK-2 cells, respectively. C) GSK-3β acetylation site prediction. D) Species conservation analysis of GSK-3β protein. The online tool for predicting GSK-3β Acetylation sites is available at GPS-PAIL 2.0 - Prediction of Acetylation on Internal Lysines (biocuckoo.org). GSK-3β protein sequences in different species were obtained from GSK3B orthologs - NCBI (nih.gov). The online Tool for species conservation analysis of GSK-3β protein was available at COBALT: Multiple Alignment Tool (nih.gov).

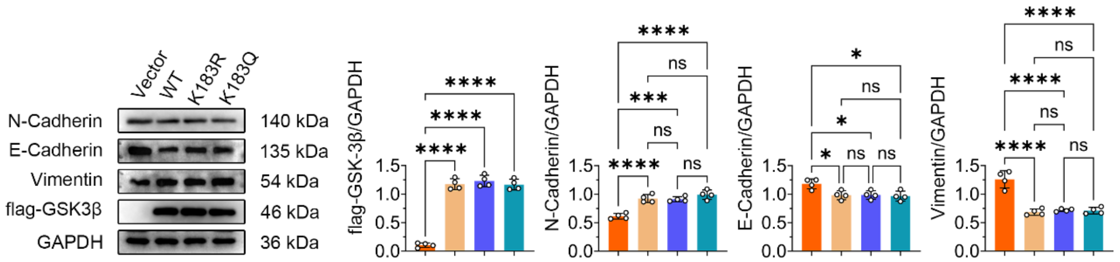

Figure S8.

GSK-3βK183 mutation has no effect on the EMT phenotype of senescent HK-2 cells. Western blots showing flag-GSK3β, N-cadherin, E-Cadherin, and Vimentin protein expression. *$p < 0.05$, **$p < 0.01$, ***$p < 0.001$, ****$p < 0.0001$, ns, no significance.

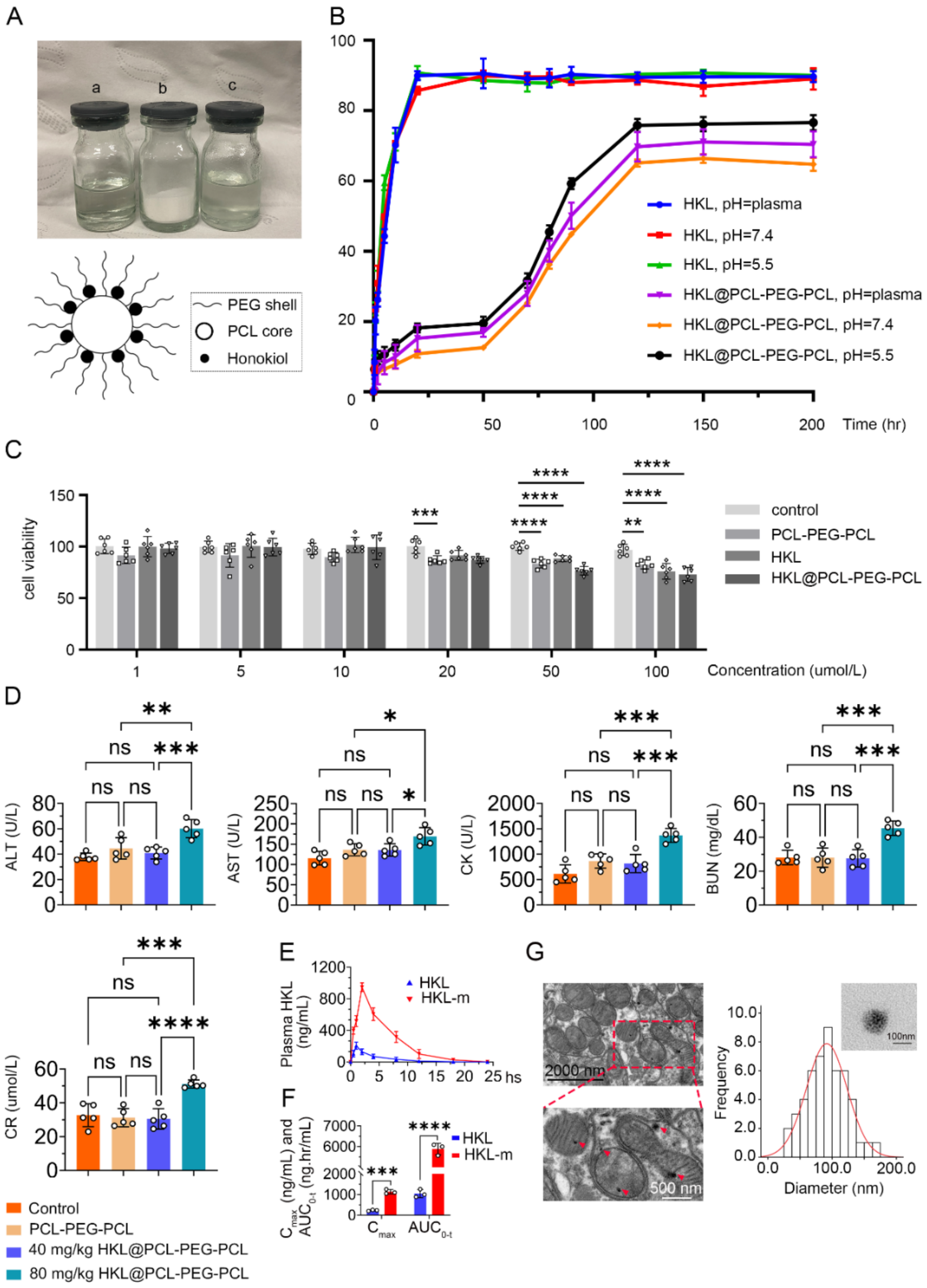

Figure S9.

In vitro release experiment and biotoxicity assessment of honokiol-micelles. A) Morphology of prepared micelles (a: PEG-PCL-PEG micelles; b: freeze-dried powder of PEG-PCL-PEG micelles; c: re-dissolved micelles solution) , and the schematic structure of prepared micelles. B) Drug release profiles of free HKL and HKL-micelles. Error bars represent the standard deviation ($n = 3$). C) HK-2 cell viability assay CCK-8. HK-2 cells were treated with various concentrations of PEG-PCL-PEG micelles for a duration of 24 hours. D) Serum chemistry tests. E) Pharmacokinetic characteristics of Honokiol in its free form and as Honokiol-micelles following oral administration. F) Maximum plasma concentration (Cmax) and total exposure (AUC) values of Honokiol in various groups. G) Micelles morphology observed by transmission electron microscopy, and particle size statistics. Red arrows indicate micelles observed in the cytoplasm and mitochondria of renal tubular epithelial cells. *$p < 0.05$, **$p < 0.01$, ***$p < 0.001$, ****$p < 0.0001$, ns, no significance.

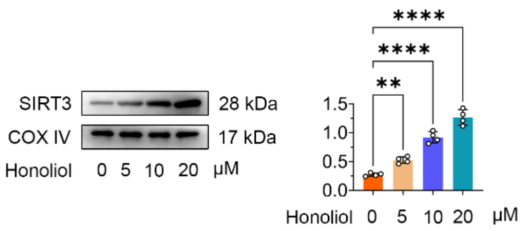

Figure S10.

Honoliol treatment increased SIRT3 expression in mitochondria of senescent HK-2 cells. $**p < 0.01$, $****p < 0.0001$

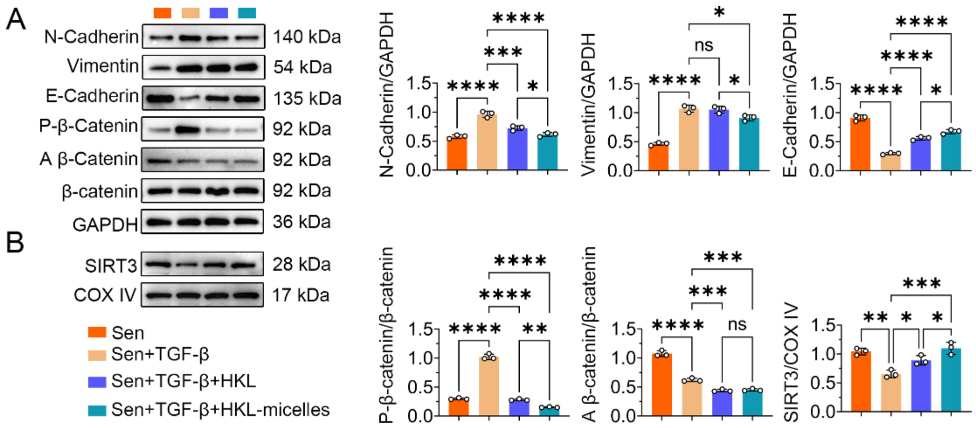

Figure S11.

Enhanced effects of HKL-micelles versus free HKL on SIRT3 expression and EMT markers in senescent HK-2 cells. A) Western blots analysis showing expression of N-cadherin, E-Cadherin, Vimentin, β-Catenin, phospho-β-Catenin-S552, non-phospho (Active) β-Catenin, and β-Catenin phosphorylated at S33/S37/T41 in senescent HK-2 cells treated with free HKL or HKL-micelles (equivalent HKL concentration). B) Western blots analysis showing mitochondrial SIRT3 expression in in senescent HK-2 cells treated with free HKL or HKL-micelles (equivalent HKL concentration). $*p < 0.05$, $**p < 0.01$, $***p < 0.001$, $****p < 0.0001$, ns, no significance. The treatment time for HKL or HKL-micelles was 24 hours, and the dose of HKL and HKL-micelles was 10 μM (HKL-equivalent dose).

Supporting Information - Tables

Table S1 Clinical specimen case data

	Gender	Age	Operation	Pathological diagnosis
Case 1	Male	38	radical nephroureterectomy	clear cell renal cell carcinoma
Case 2	Female	37	radical nephroureterectomy	clear cell renal cell carcinoma
Case 3	Male	39	radical nephroureterectomy	clear cell renal cell carcinoma
Case 4	Male	36	radical nephroureterectomy	clear cell renal cell carcinoma
Case 5	Female	37	radical nephroureterectomy	clear cell renal cell carcinoma
Case 6	Male	33	radical nephroureterectomy	clear cell renal cell carcinoma
Case 7	Male	63	radical nephroureterectomy	clear cell renal cell carcinoma
Case 8	Female	70	radical nephroureterectomy	clear cell renal cell carcinoma
Case 9	Male	66	radical nephroureterectomy	clear cell renal cell carcinoma
Case 10	Male	68	radical nephroureterectomy	clear cell renal cell carcinoma
Case 11	Male	61	radical nephroureterectomy	clear cell renal cell carcinoma
Case 12	Female	65	radical nephroureterectomy	clear cell renal cell carcinoma

Table S2 Primers for RT-qPCR

	forward	reverse
SIRT3 (m)	GCCCAATGTCACTCACTACTTCCTG	TCCCAGATGCTCTCTCAAGCCCGTC
SIRT3(h)	AGCTCGGCATCTGTTGGTTAC	GCACCAGGGCCTCAAGACTAC
E-cadherin(h)	CTGTGCCCAGCCTCCATGTTTT	CTGGATAGCTGCCCATTGCAAGTTA
N-Cadherin(h)	GTGCATGAAGGACAGCCTCT	CCACCTTAAAATCTGCAGGC
Vimentin(h)	AAATGGCTCGTCACCTTCGT	TTGCGCTCCTGAAAAACTGC
c-MYC(h)	TAGTGGAAAACCAGCAGCCTC	AAGTTCTCCTCCTCGTCGCA
Cyclin D1(h)	GCATGTTCGTGGCCTCTAAGA	CGGTGTAGATGCACAGCTTCTC
GAPDH(h)	ACAACTTTGGTATCGTGGAAGG	GCCATCACGCCACAGTTTC
GAPDH(m)	GGAGCGAGATCCCTCCAAAAT	GGCTGTTGTCATACTTCTCATGG

Table S3 Primers for SIRT3 MSP

	forward	reverse
methylated primer	GAGTTTTTTTAGACGGTGTTATATAGC	CGAAAATAAATAAACATTTCCGAC
unmethylated primer	TTTTTTAGATGGTGTTATATAGTGG	CCAAAAATAAATAAACATTTCCAAC
internal control	CCAACTCCAAATCCCCTCTCTAT	TGATTAATTTAGATTGGGTTTAGAGAAGGA.

Supporting Information - Genotype identification report

1. Primer Information

Strain Name	Primer Number	Primer Sequence(5'→3')	Primer Type	Reactivity Group	Product Length
**sirt3-flox**	YDS0743-1A_ sirt3-flox	CATACTTGGCTTTGTTTGTTTAGA	Forward	A	WT = 229 bp
	YDS0743-2A_ sirt3-flox	CCCAGGGCCGTTTATGC	Reverse	A	Mutant = 381 bp
**cdh16-cre**	YDS0122-1A_Cdh16_M420	GCAGATCTGGCTCTCCAAAG	Forward	A	Mutant = 420 bp
	YDS0122-2A_Cdh16_W0	AGGCAAATTTTGGTGTACGG	Reverse	A	WT = 0

2. Identification information

Test Date	Tester	Date of Submission	Client	Strain	Sample Number	Quantity
2023-04	YHZ	2023-04	**Wang Jing**	**sirt3-flox/cdh16-cre**	#1-42	42

3. Identification Results

Gene	Homozygous HO	Hybrid HZ	Wild WT	Transgenic Tg	To be Reviwed
**sirt3-flox**	1-23,28,30,31,35-37,40,41	24-27,29,32,33,34,38,39,42	/	/	/
**cdh16-cre**	/	/	1,4,6,8,10-12,14,15,17,18,20 -22,25,26,29,32,33,35,37-39, 42	2,3,5,7,9,13,16,19,23,24,27,28,30,31,34,36,40,41	/

Agarose gel electrophoresis

1. Agarose concentration: 2%
2. Electrophoresis solution: 0.5X TBE
3. Electrophoresis parameters: 180 V, 400 mA, 40 min (duration can be adjusted according to the fragment size)

The imaging results after agarose gel electrophoresis were processed as follows:

M: All-Gold Trans DNA Marker Il (BM411-02)

H2O: No template control group, ddH20 was used instead of DNA template

B6: Wild type control, using C57BL/6J mouse tail DNA as template

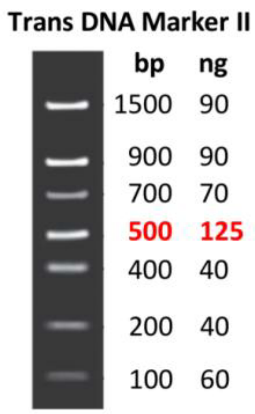

4 . PCR System

Reaction Component	Volume (μL)
ddH$_2$O	8
2×Taq Master Mix (Vazyme Cat#P112-AA)	10
Forward Primer (10pmol/μL)	0.5
Reverse Primer (10pmol/μL)	0.5
Genomic DNA	1
**Total**	**20**

5. PCR Procedure

Step	Temperature	Time	Note
1	95 °C	5 min	
2	95 °C	20 sec	Step3: -0.5 C per cycle decrease.<br>Repeat steps 2-4 for 10 cycles (Touchdown)
3	60 °C	20 sec	
4	68 °C	1 min	
5	95 °C	20 sec	Repeat steps 5-7 for 26 cycles
6	55 °C	20 sec	
7	72 °C	1 min	
8	72 °C	5 min	
9	10 °C	hold	

A

SIRT3-flox_Primer A-mix

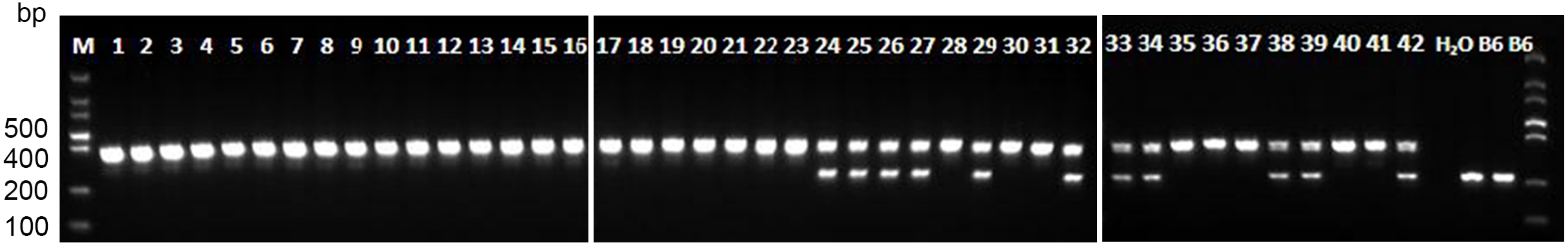

B

CDH-16 Cre_Primer A-mix

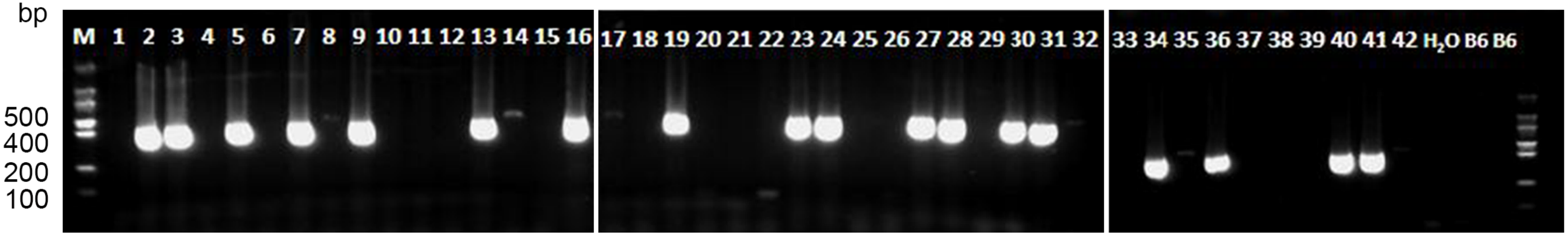

Supporting Information - RNA extraction library construction and sequencing (RNA-seq)

1. RNA sample detection

Kidney samples (each 3-5 mice in the young_UUO group vs. old_UUO group) were collected for mRNA expression analyses. Total RNA was extracted as described above, and the total RNA quantity and purity were analyzed of NanoDrop™ One/OneC and Qubit™ RNA HS Assay Kit (Thermofisher Scientific, USA), high-quality RNA samples with RIN value > 7.0 (Agilent 4200 TapeStation) were used to construct sequencing library.

2. Library construction

After extraction of total RNA, mRNA was purified from total RNA (5ug) using Dynabeads Oligo (DT) (Thermo Fisher, CA, USA) with two rounds of purification. Then, the cleaved RNA fragments were reverse-transcribed to obtain the first strand of cDNA by M-MuLV Reverse Transcriptase (Thermofisher Scientific, USA), which were next used to synthesize U-labeled second-stranded DNAs with E. coli DNA polymerase I (NEB, cat.m0209, USA), RNase H (NEB, cat.m0297, USA) and dUTP Solution (Thermo Fisher, cat.R0133, USA). An A-base was then added to the blunt ends of each strand, preparing them for ligation to the indexed adapters. Each adapter contained a T-base overhang for ligating the adapter to the A-tailed fragmented DNA. Dual-index adapters were ligated to the fragments, and size (about 200 bp) selection was performed with AMPureXP beads. After the heat-labile UDG enzyme (NEB, cat.m0280, USA) treatment of the U-labeled second-stranded DNAs, the ligated products were amplified with PCR and pure with AMPureXP beads again to obtain the final cDNA library. The average insert length for the final cDNA library was detected by Kapa qPCR, Agilent 4200 TapeStation. Finally, according to the manufacturer's protocol, we performed the paired-end sequencing (PE150) on an Illumina Novaseq™ 6000 (LC-Bio Technology CO., Ltd., Ltd., Hangzhou, China).

3. Sequencing data preprocessing

Reads obtained from the sequencing machines include raw reads containing adapters or low-quality bases, affecting the following assembly and analysis. Thus, a fastp preprocessor further filters read to get high-quality clean reads. The parameters were as follows:1) removing reads containing adapters; 2) removing reads containing polyA and polyG; 3) removing reads containing more than 5% of unknown nucleotides (N); 4) removing low-quality reads containing more than 20% of low-quality (Q-value⩽20) bases. The sequence quality was verified using FastQC (http://www.bioinformatics.babraham.ac.uk/projects/fastqc), including the clean data's Q20, Q30, and GC content. HISAT2 (Hierarchical Indexing for Spliced Alignment of Transcripts) Software was selected to compare the transcriptome sequencing Reads to the reference genome.

4. Data analysis

Genes differential expression analysis was performed by DESeq2 software. The genes with q<0.05 and absolute fold change>1 were considered differentially expressed. Gene ontology (GO) categories and Kyoto Encyclopedia of Genes and Genomes (KEGG) analysis The bioinformatics analysis for RNA sequencing was performed using OmicStudio tools (http://www.omicsudio.cn/tool). Gene Ontology (GO) functional enrichment analysis (http://www.geneontology.org/) and Kyoto Encyclopedia of Genes and Genomes (KEGG) enrichment analysis (https://www.genome.jp/kegg/) were used to analyze the biological functions of the predicted target genes.

Supporting Information - Synthesis and characterization of the PEG–PCL–PEG copolymers

Materials and methods

In this procedure, polyethylene glycol (PEG) served as the macroinitiator and stannous octoate ($Sn(Oct)_2$) as the catalyst. A precisely measured mixture containing ε-caprolactone monomer (2 g), PEG macromer (2 g), and $Sn(Oct)_2$ catalyst (0.01 mmol) was polymerized at 120°C for 12 hours under an inert atmosphere. After polymerization, the viscous product was cooled to room temperature, purified by dissolution in chloroform (50 mL) followed by precipitation with ice-cold diethyl ether (-20°C), and subsequently vacuum-dried at 25°C for 24 hours to remove residual solvents. To prepare HKL-loaded micelles, PEG-PCL-PEG copolymer (20 mg) and varying amounts of HKL (5 mg) were dissolved in 2 mL of anhydrous acetone. This solution was added dropwise to 25 mL of deionized water using a syringe pump (0.5 mL/min) under vortex mixing (800 rpm). The mixture was then stirred magnetically for 24 h at 25 °C to allow solvent evaporation and micelle maturation. Unencapsulated HKL was removed by filtration through a 0.45 μm cellulose acetate membrane. The micelles were collected by centrifugation (20,000 × g, 20 min, 4°C) and subsequently lyophilized for 48 hours (-80°C, 14 Pa) to obtain a powder.

Results

The structure and composition of the synthesized PEG–PCL–PEG copolymers were characterized by Proton Nuclear Magnetic Resonance Spectroscopy (NMRS) at 400 MHz in deuterated chloroform. The presence of ethylene (CH2) in PCL was identified at roughly 1.2, 1.6, 2.3, and 4.1 ppm, whereas the methylene (CH2) groups of PEG were detected near 3.7 ppm. The FT-IR spectrum of the PEG–PCL–PEG copolymer showed significant peaks at 1722 and 1106 cm^{-1}. These can be associated with the existence of carboxylic ester (C=O) and ether (C-O) functional groups within the structure. The weight- and number-average molecular weights of the copolymer were measured by gel permeation

chromatography (GPC, Knaure, Germany). The results showed that the copolymer's average molecular weights were around 15.4±4.7 kDa and 14.1±4.2 kDa, respectively (see Table 1 for details). The thermal characteristics of the tri-block copolymers were analyzed through differential scanning calorimetry (DSC, Mettler Toledo model Star SW 9.30, Selangor, Switzerland). A notable endothermic peak was detected at a temperature of 55.3 °C. The results offer proof of the effective creation of the PEG–PCL–PEG copolymer. As illustrated in Table 1, it presents the features of the synthesized copolymers.

Table 1 Molecular characterics of the synthesized copolymers

Mn (Da)	Mw (Da)	Pdl	Tm/°C	DP_{PEG}	DP_{PCL}
14156.2±4239.9	15432±4703.84	1.086±0.025	60.06±1.012	136.36	82.63±10.21

Mw/Mn=Polydispersity index of the polymers (Pdl) determined by GPC analysis. DP: degree of polymerization.

Figure 1G

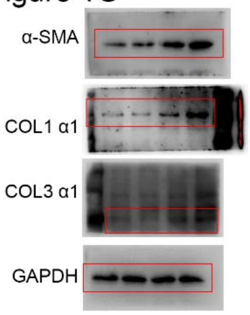

Figure 2A

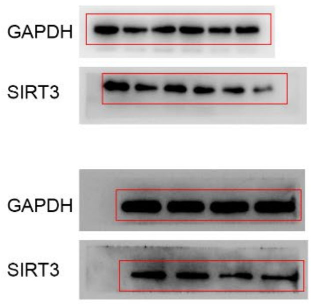

Figure 2F

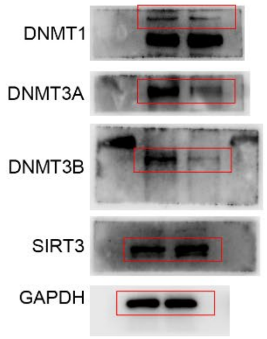

Figure 3D

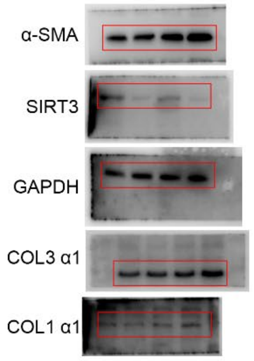

Figure 3H

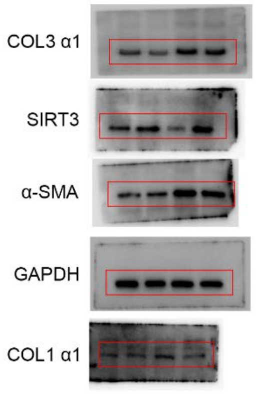

Figure4F

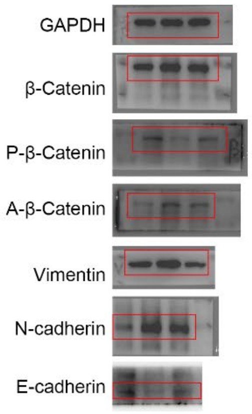

Figure 5B

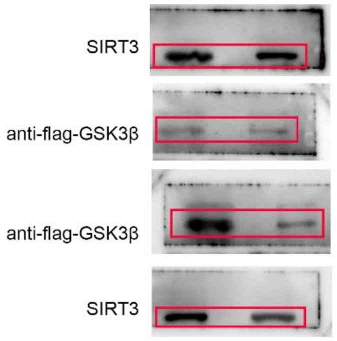

Figure 5C

Figure 5D

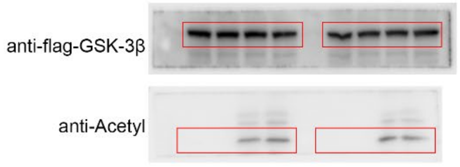

Figure 5E

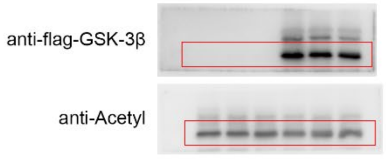

Figure 5F

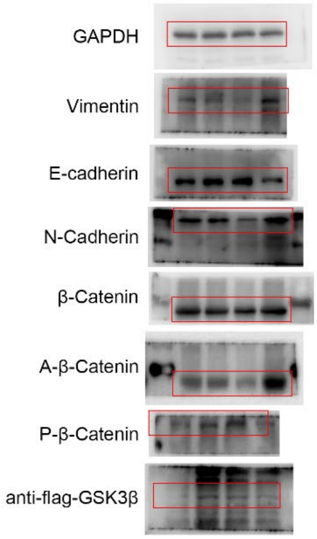

Figure 6H

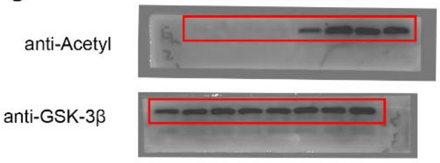

Figure 6I

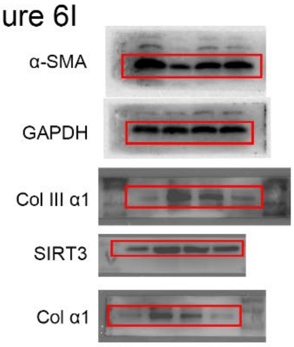

Figure 6J

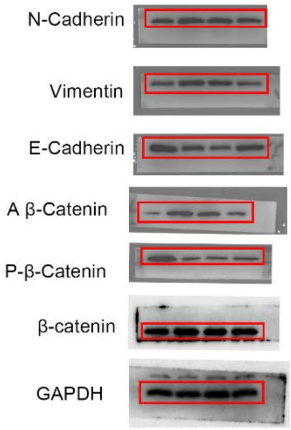

Figure 2D

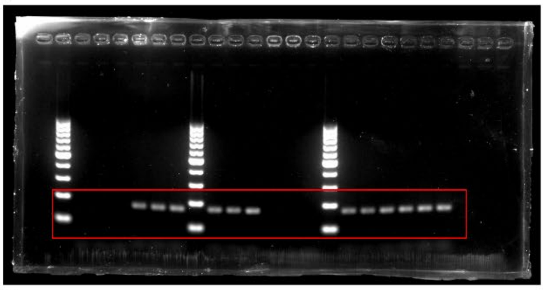

Figure 2D

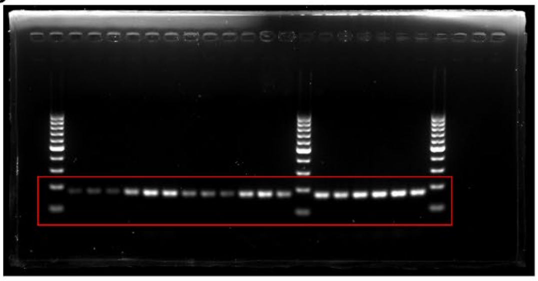

Figure 6K

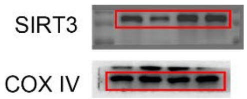

Figure S2C

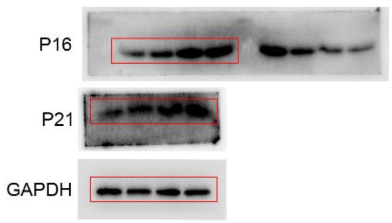

Figure S3B

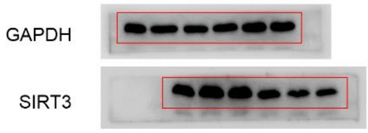

Figure S5B

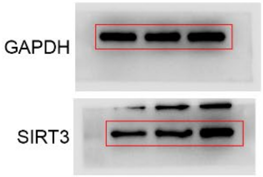

Figure S5E

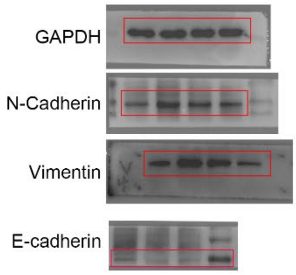

Figure S7A

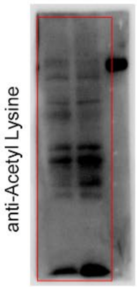

Figure S7B

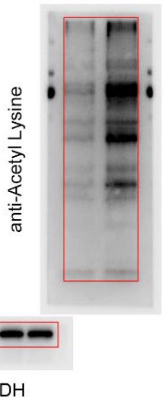

Figure S8

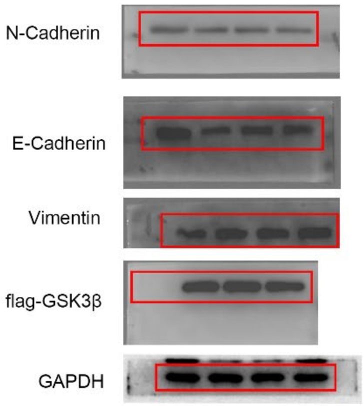

Figure S11

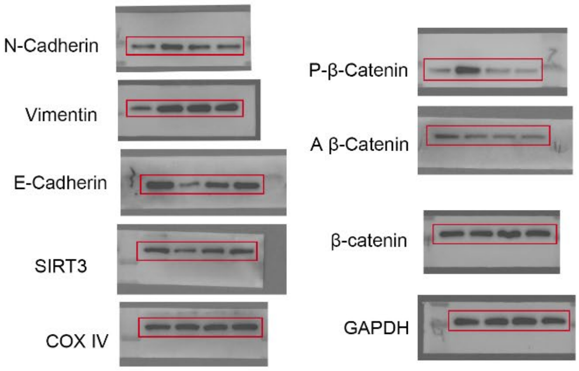

Figure S10

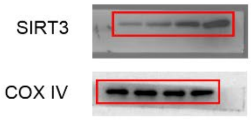

Supplement: Supplementary file 1 — Supporting Information [file ADVS-12-e17248-s001.pdf]
